# Supplementary figures and images for: Phenotypic and molecular evolution across 10,000 generations in laboratory budding yeast populations
Source: eLife. 2021 Jan 19;10:e63910. doi: 10.7554/eLife.63910 (PMC7815316; doi:10.7554/eLife.63910)

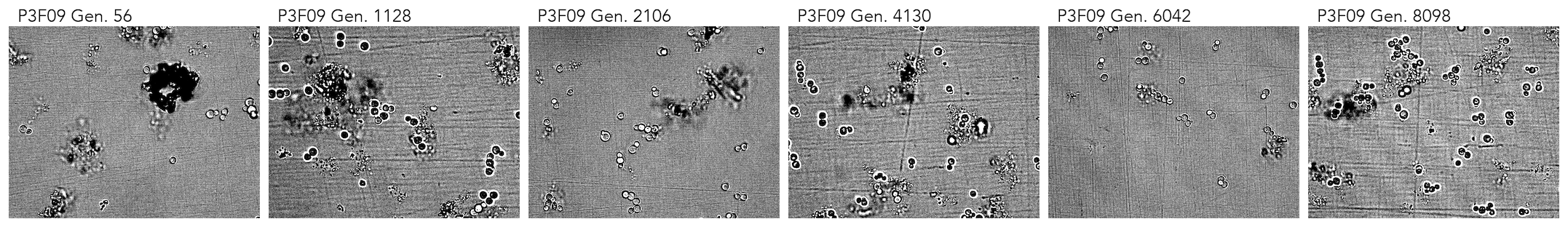

Supplement: Supplementary file 6. [file elife-63910-supp6.zip › imaging/cropped_P3F09.png]

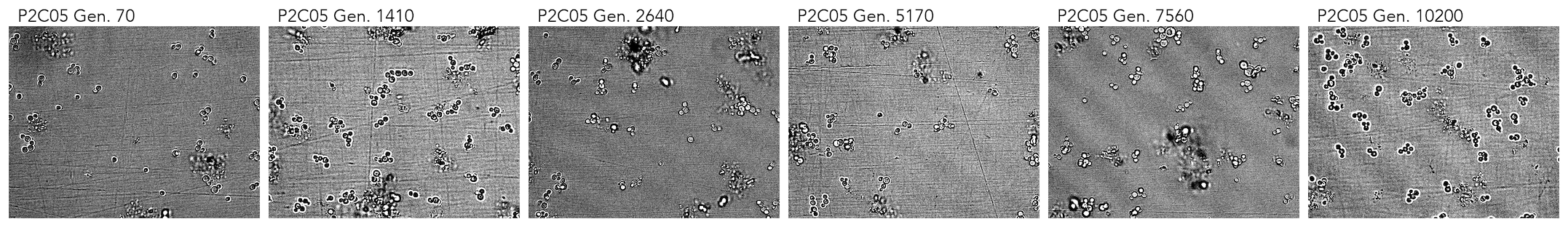

Supplement: Supplementary file 6. [file elife-63910-supp6.zip › imaging/cropped_P2C05.png]

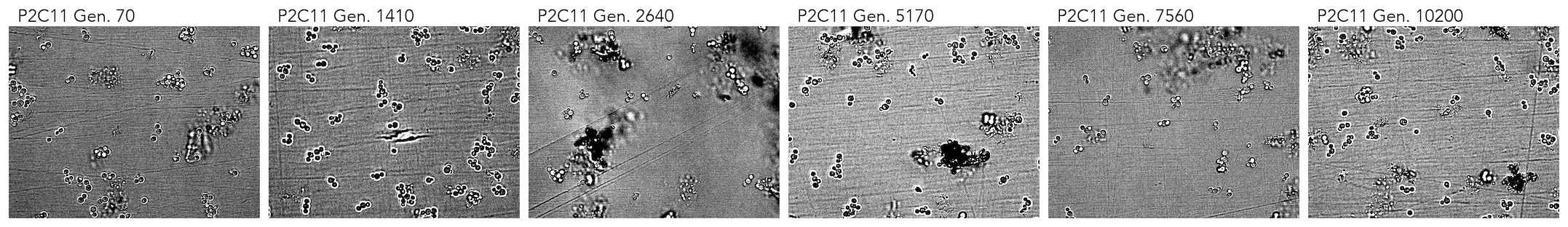

Supplement: Supplementary file 6. [file elife-63910-supp6.zip › imaging/cropped_P2C11.png]

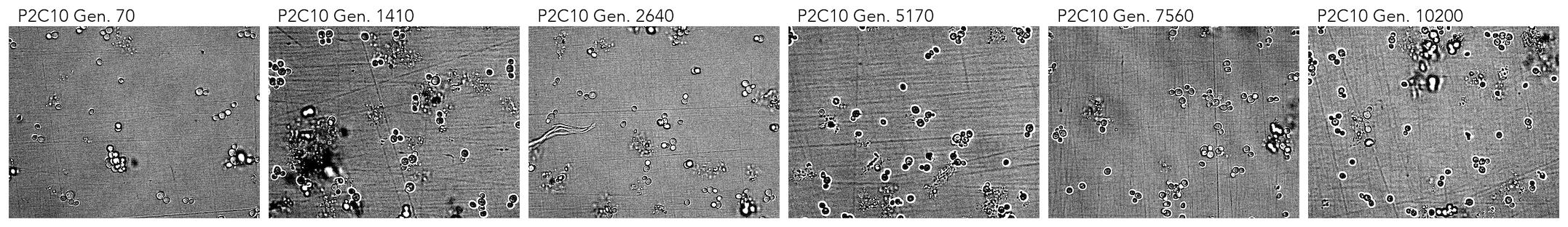

Supplement: Supplementary file 6. [file elife-63910-supp6.zip › imaging/cropped_P2C10.png]

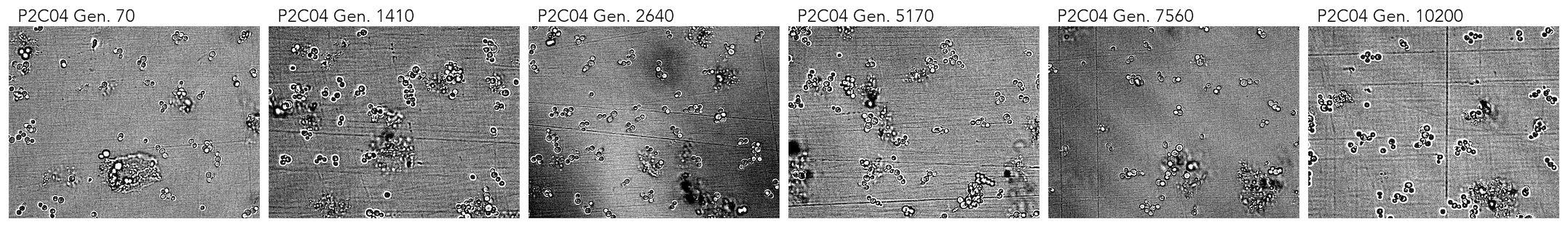

Supplement: Supplementary file 6. [file elife-63910-supp6.zip › imaging/cropped_P2C04.png]

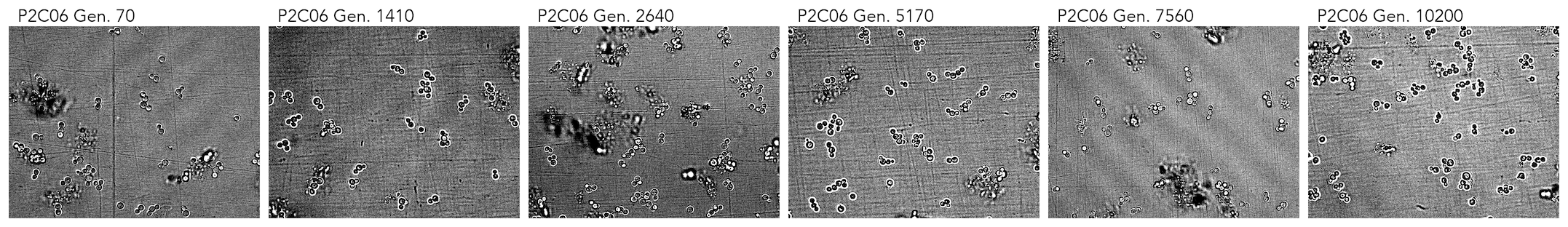

Supplement: Supplementary file 6. [file elife-63910-supp6.zip › imaging/cropped_P2C06.png]

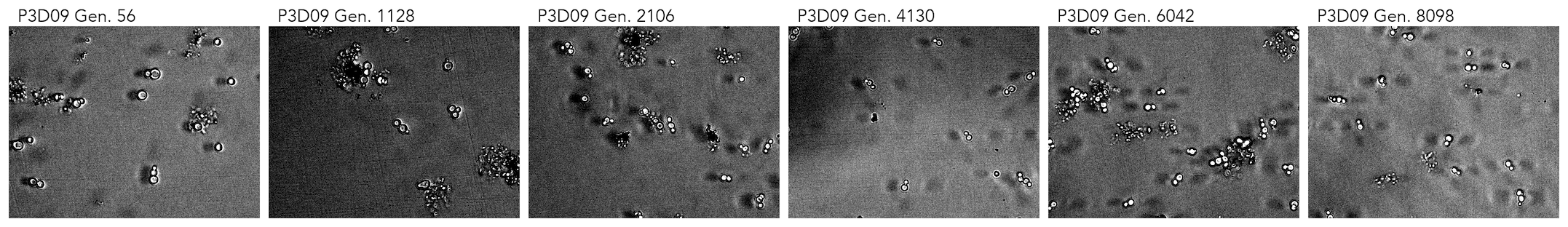

Supplement: Supplementary file 6. [file elife-63910-supp6.zip › imaging/cropped_P3D09.png]

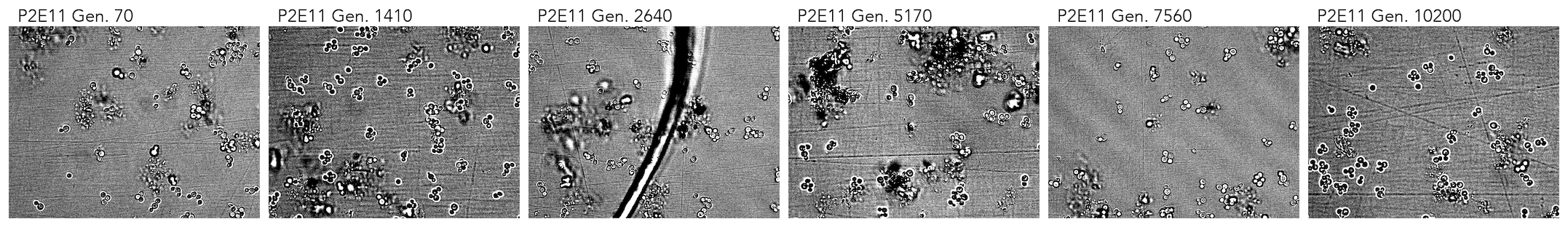

Supplement: Supplementary file 6. [file elife-63910-supp6.zip › imaging/cropped_P2E11.png]

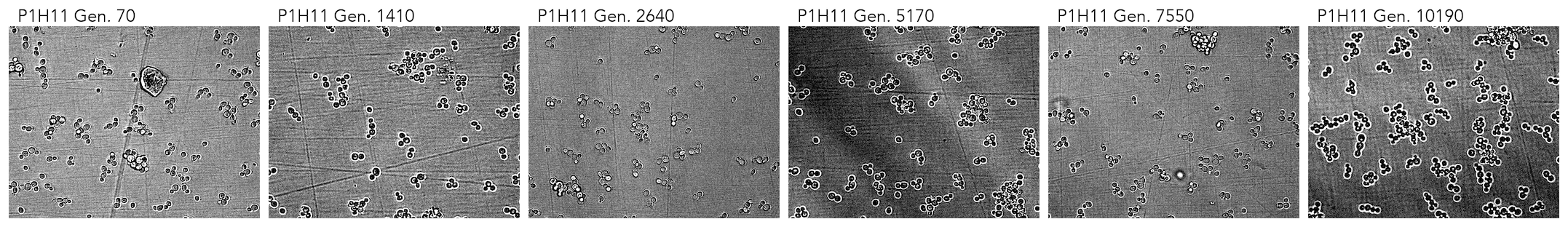

Supplement: Supplementary file 6. [file elife-63910-supp6.zip › imaging/cropped_P1H11.png]

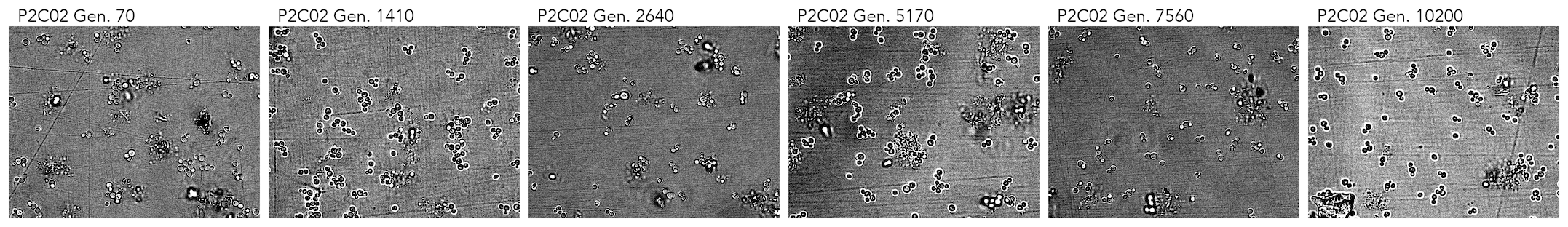

Supplement: Supplementary file 6. [file elife-63910-supp6.zip › imaging/cropped_P2C02.png]

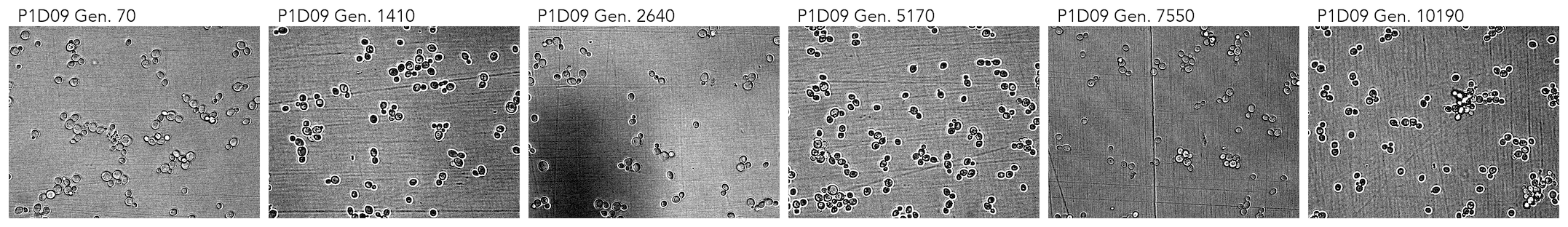

Supplement: Supplementary file 6. [file elife-63910-supp6.zip › imaging/cropped_P1D09.png]

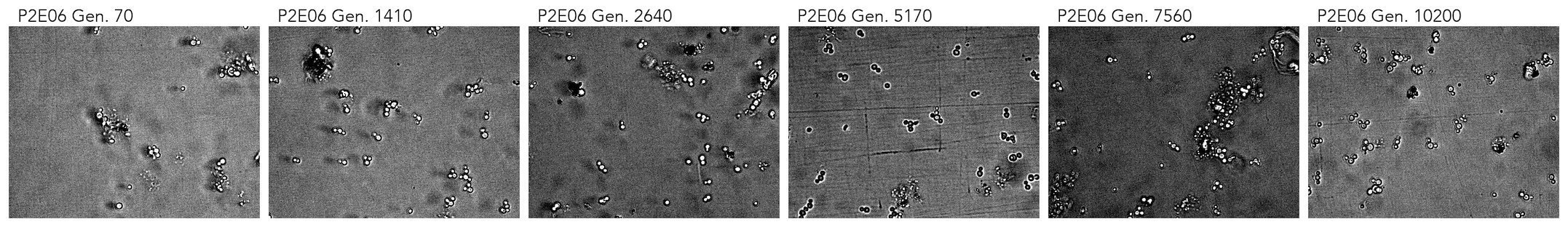

Supplement: Supplementary file 6. [file elife-63910-supp6.zip › imaging/cropped_P2E06.png]

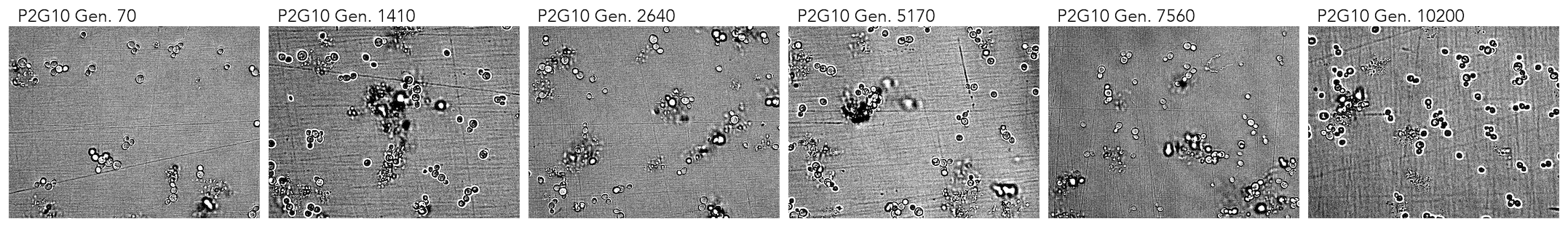

Supplement: Supplementary file 6. [file elife-63910-supp6.zip › imaging/cropped_P2G10.png]

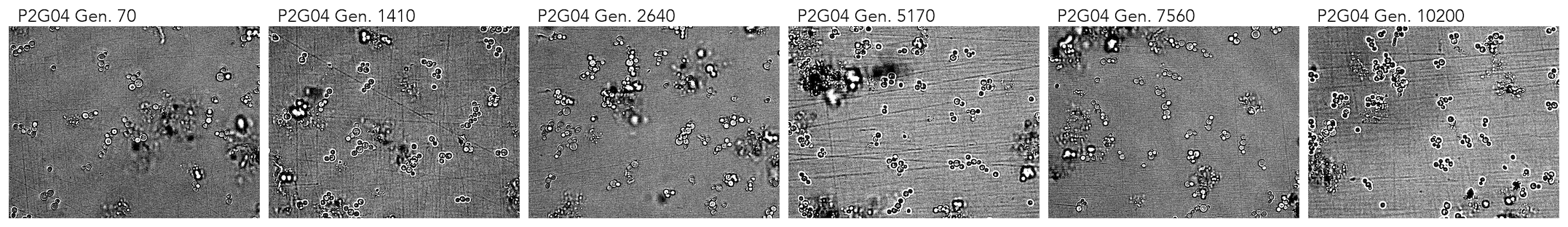

Supplement: Supplementary file 6. [file elife-63910-supp6.zip › imaging/cropped_P2G04.png]

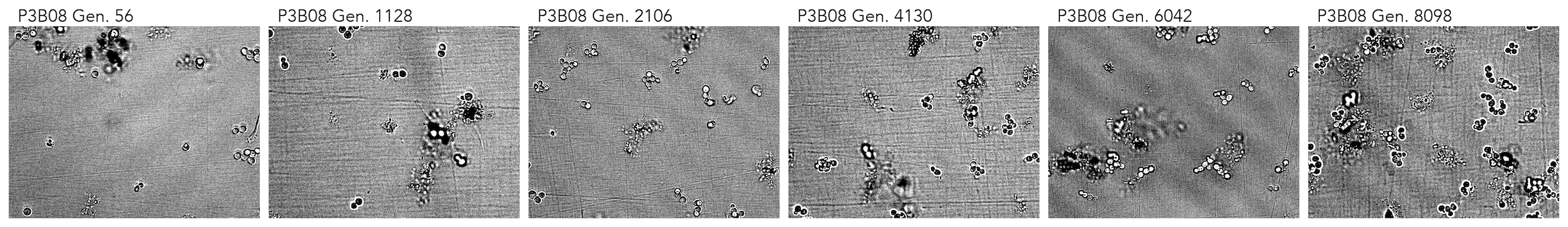

Supplement: Supplementary file 6. [file elife-63910-supp6.zip › imaging/cropped_P3B08.png]

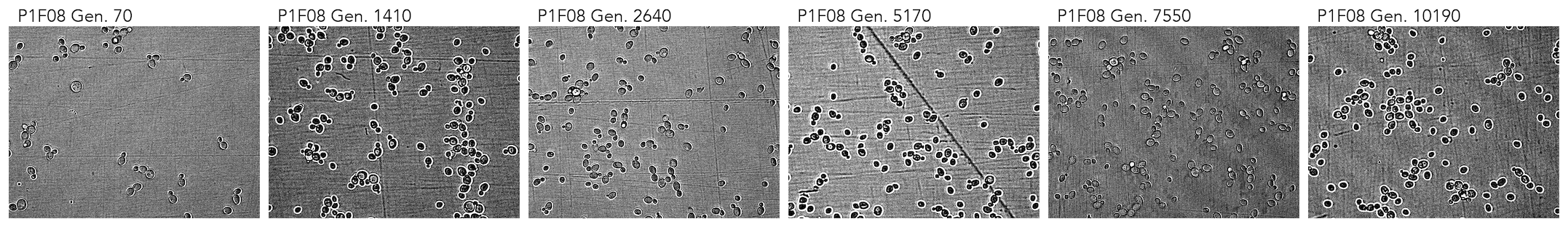

Supplement: Supplementary file 6. [file elife-63910-supp6.zip › imaging/cropped_P1F08.png]

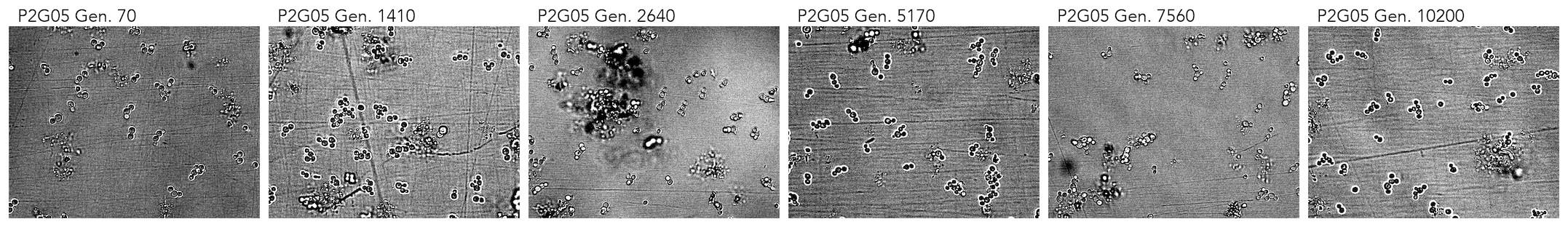

Supplement: Supplementary file 6. [file elife-63910-supp6.zip › imaging/cropped_P2G05.png]

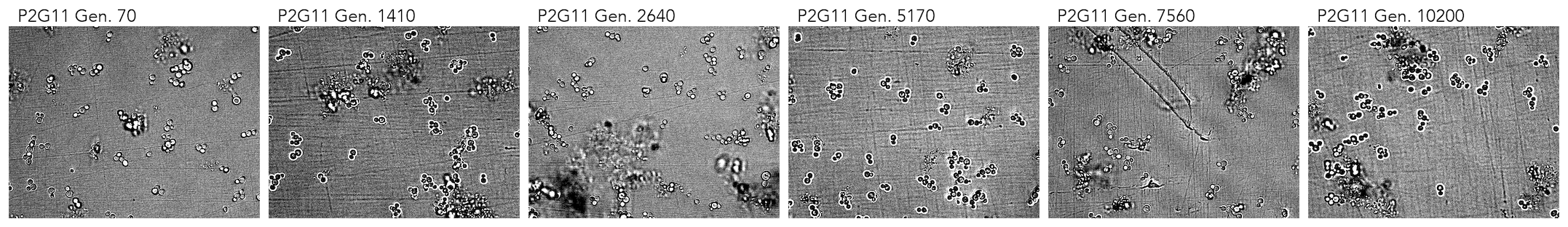

Supplement: Supplementary file 6. [file elife-63910-supp6.zip › imaging/cropped_P2G11.png]

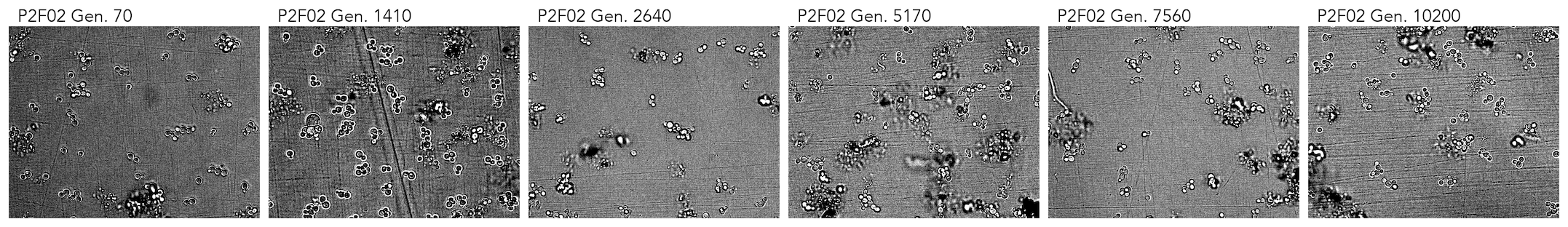

Supplement: Supplementary file 6. [file elife-63910-supp6.zip › imaging/cropped_P2F02.png]

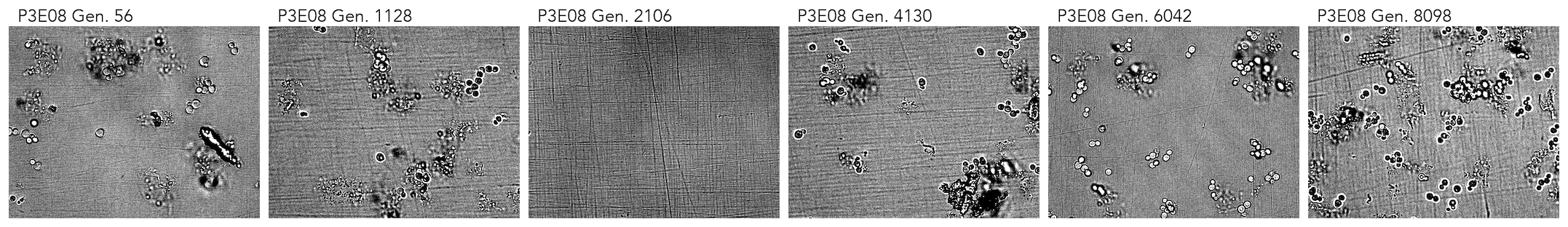

Supplement: Supplementary file 6. [file elife-63910-supp6.zip › imaging/cropped_P3E08.png]

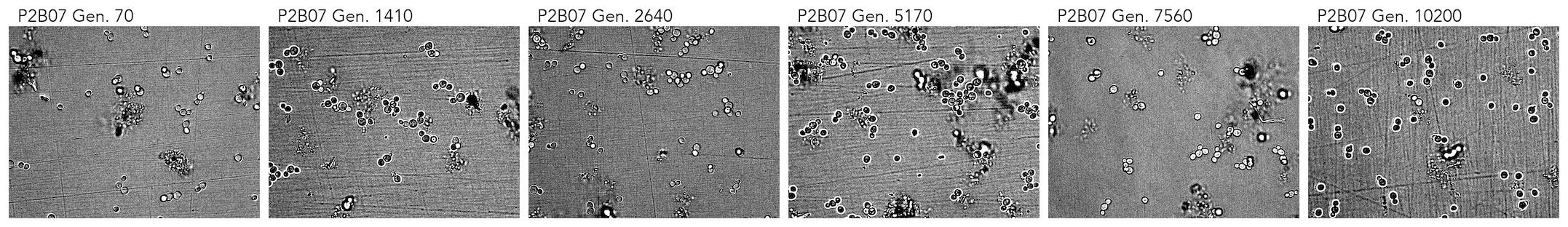

Supplement: Supplementary file 6. [file elife-63910-supp6.zip › imaging/cropped_P2B07.png]

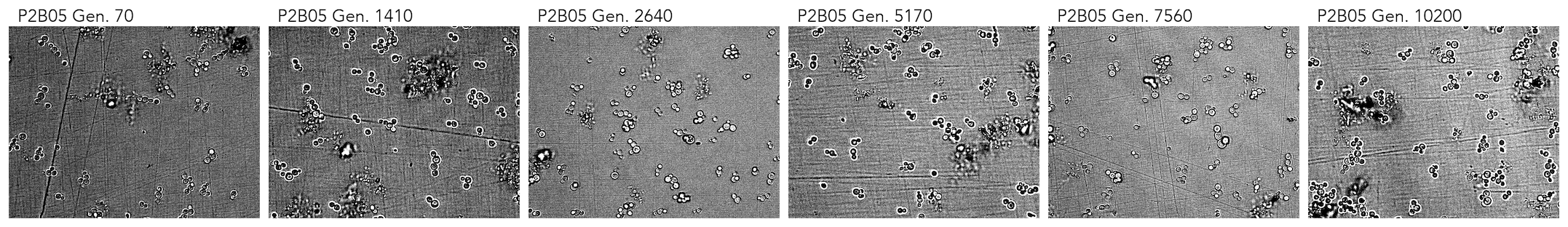

Supplement: Supplementary file 6. [file elife-63910-supp6.zip › imaging/cropped_P2B05.png]

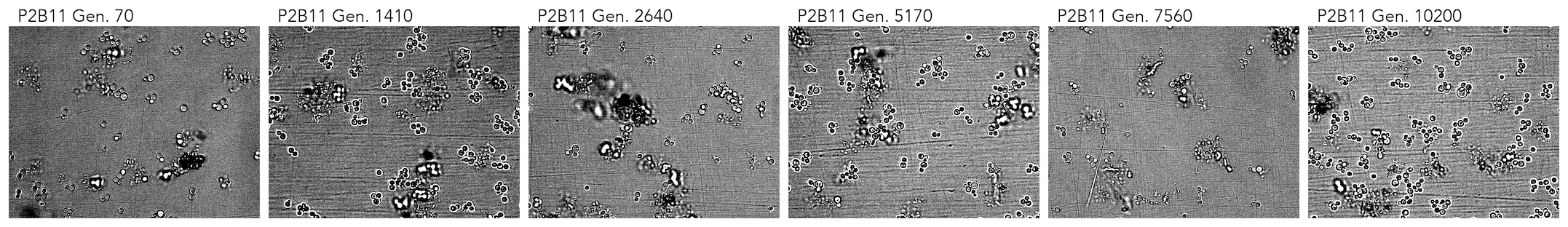

Supplement: Supplementary file 6. [file elife-63910-supp6.zip › imaging/cropped_P2B11.png]

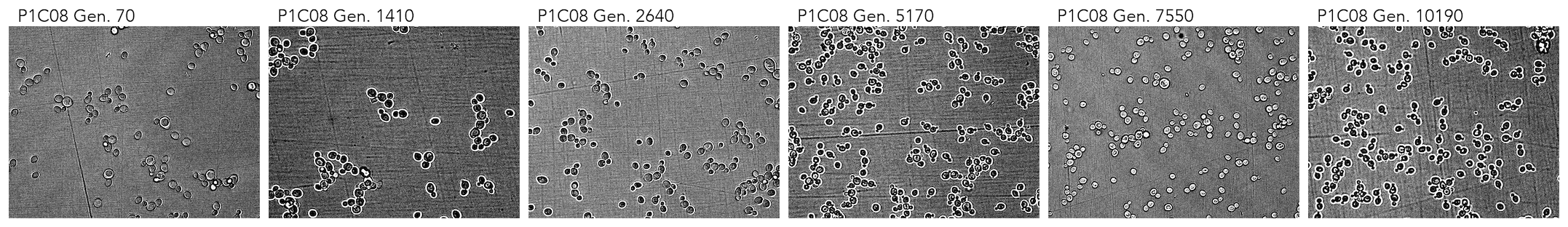

Supplement: Supplementary file 6. [file elife-63910-supp6.zip › imaging/cropped_P1C08.png]

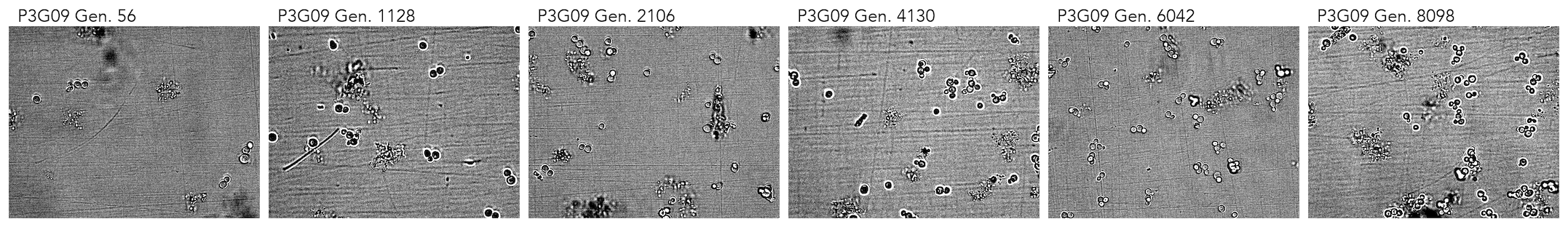

Supplement: Supplementary file 6. [file elife-63910-supp6.zip › imaging/cropped_P3G09.png]

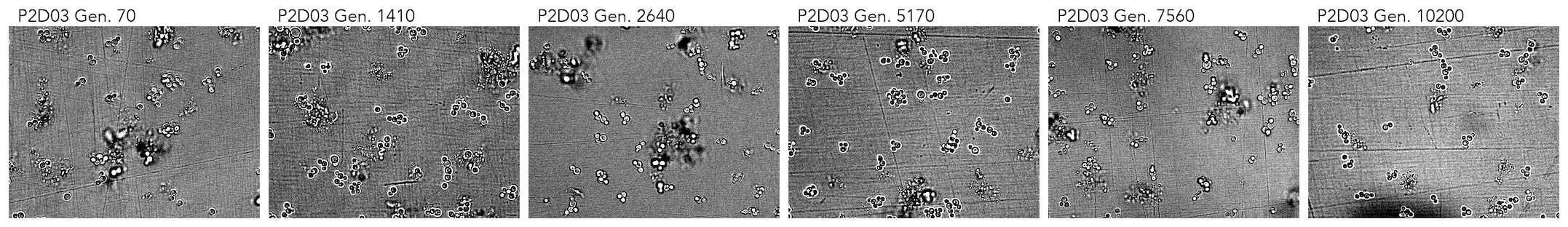

Supplement: Supplementary file 6. [file elife-63910-supp6.zip › imaging/cropped_P2D03.png]

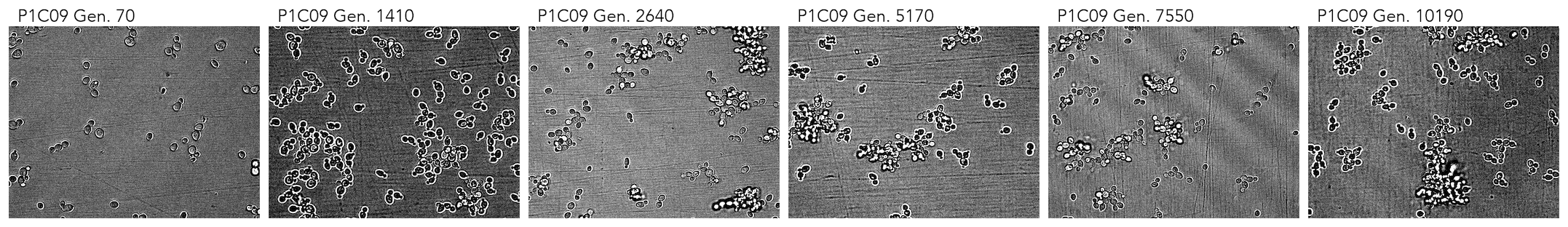

Supplement: Supplementary file 6. [file elife-63910-supp6.zip › imaging/cropped_P1C09.png]

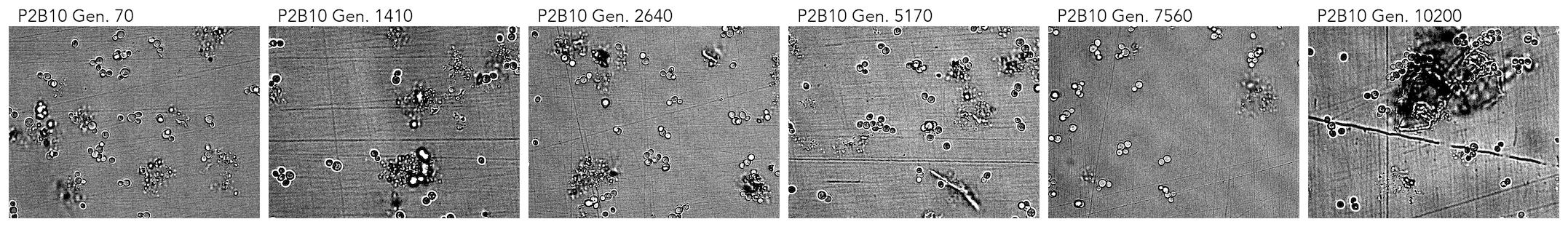

Supplement: Supplementary file 6. [file elife-63910-supp6.zip › imaging/cropped_P2B10.png]

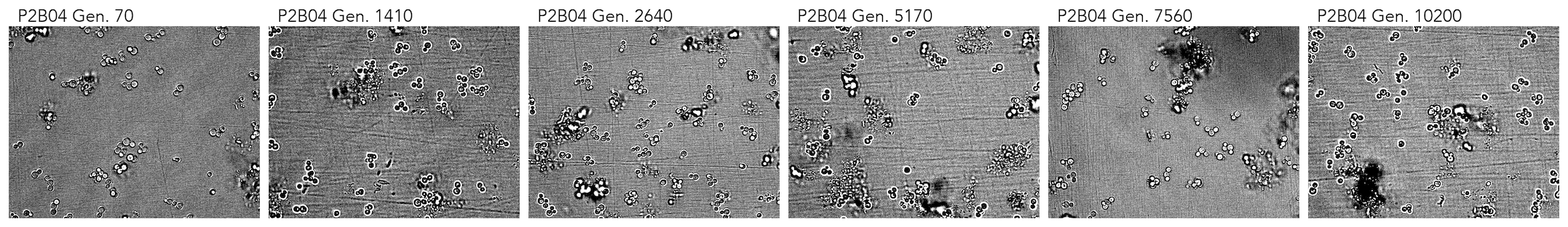

Supplement: Supplementary file 6. [file elife-63910-supp6.zip › imaging/cropped_P2B04.png]

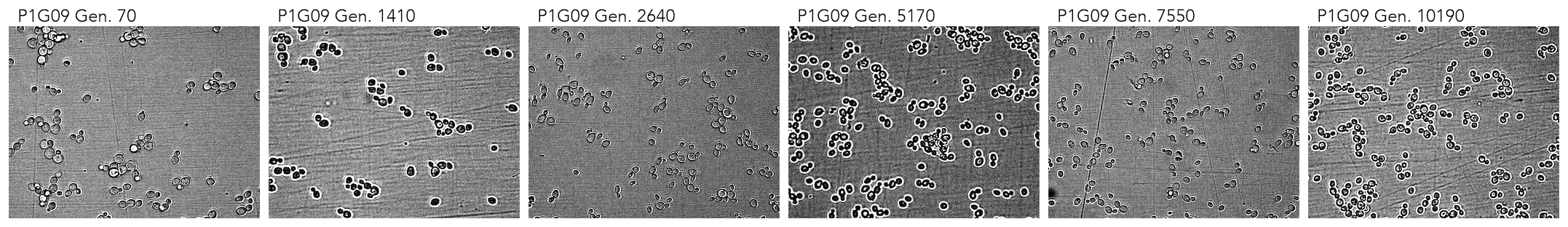

Supplement: Supplementary file 6. [file elife-63910-supp6.zip › imaging/cropped_P1G09.png]

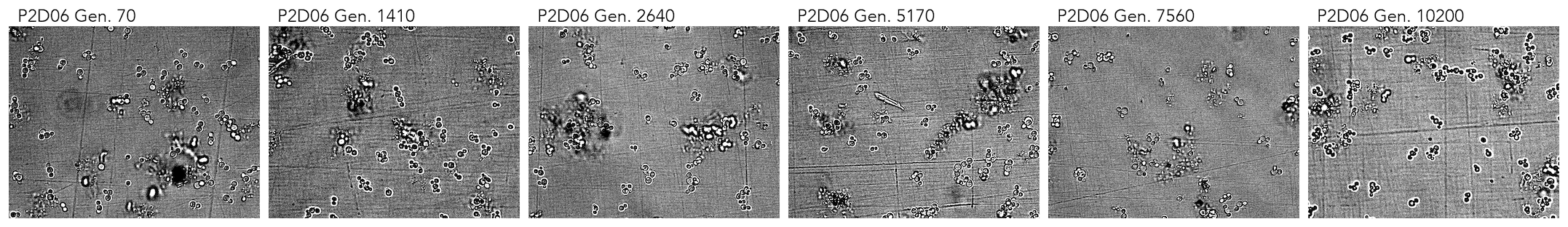

Supplement: Supplementary file 6. [file elife-63910-supp6.zip › imaging/cropped_P2D06.png]

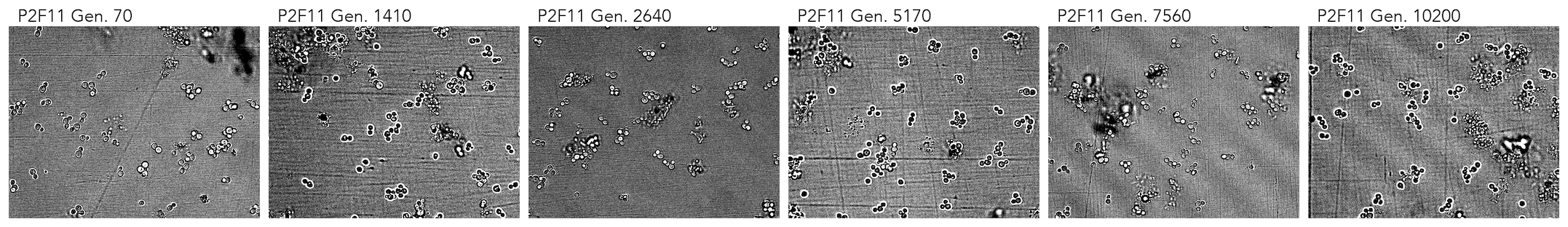

Supplement: Supplementary file 6. [file elife-63910-supp6.zip › imaging/cropped_P2F11.png]

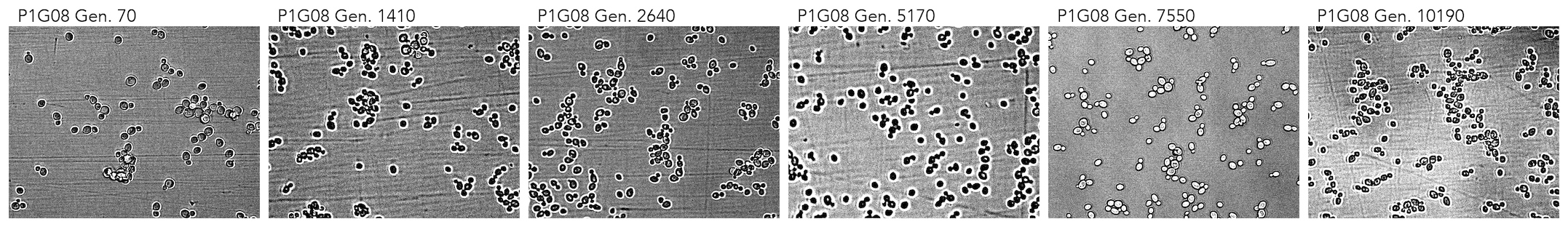

Supplement: Supplementary file 6. [file elife-63910-supp6.zip › imaging/cropped_P1G08.png]

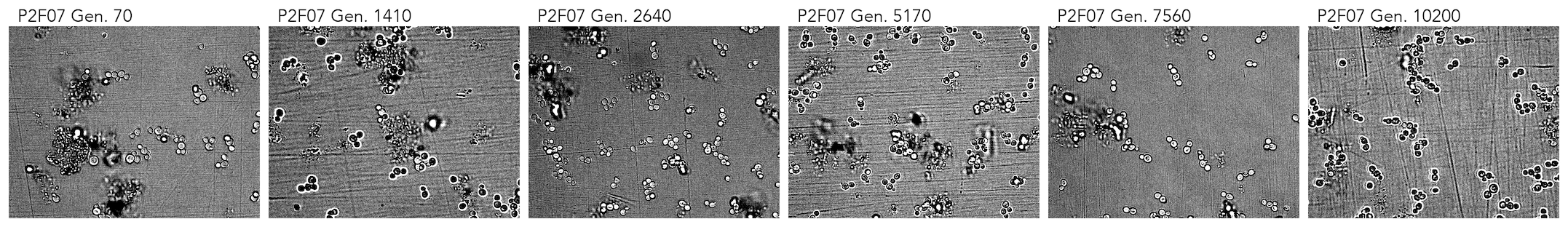

Supplement: Supplementary file 6. [file elife-63910-supp6.zip › imaging/cropped_P2F07.png]

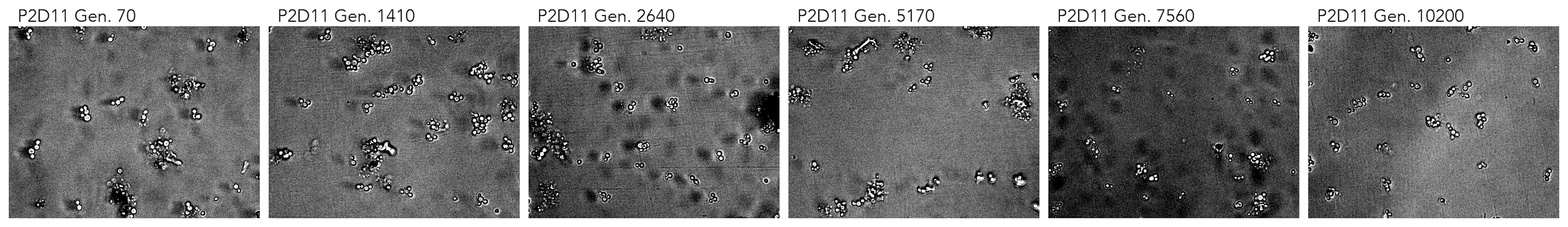

Supplement: Supplementary file 6. [file elife-63910-supp6.zip › imaging/cropped_P2D11.png]

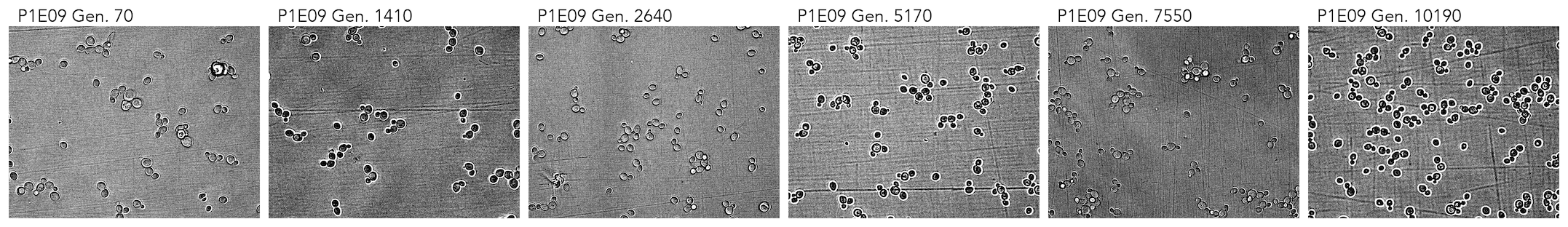

Supplement: Supplementary file 6. [file elife-63910-supp6.zip › imaging/cropped_P1E09.png]

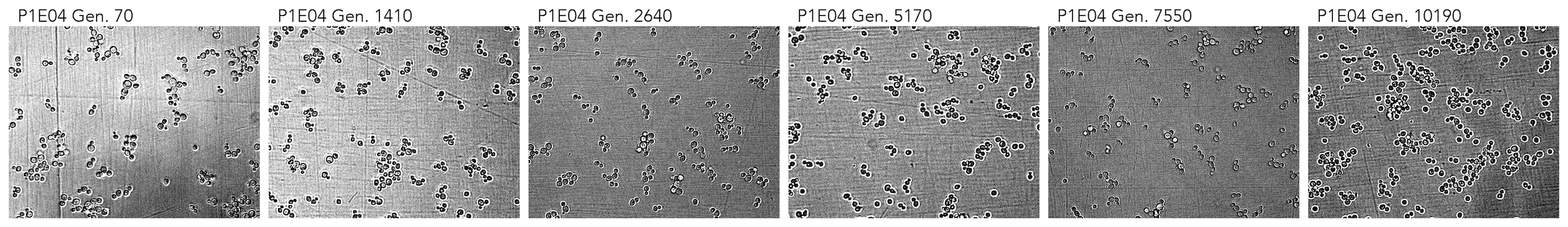

Supplement: Supplementary file 6. [file elife-63910-supp6.zip › imaging/cropped_P1E04.png]

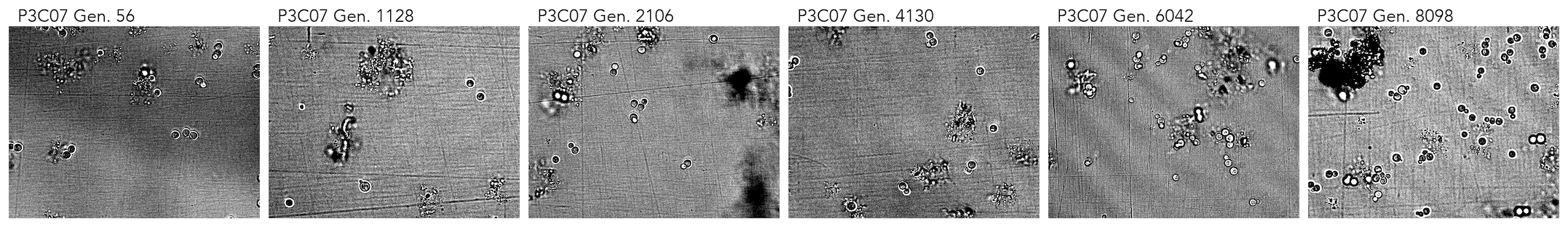

Supplement: Supplementary file 6. [file elife-63910-supp6.zip › imaging/cropped_P3C07.png]

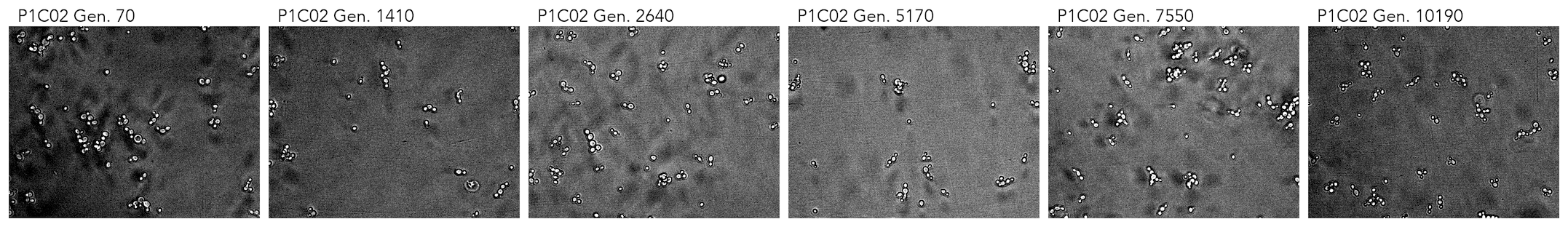

Supplement: Supplementary file 6. [file elife-63910-supp6.zip › imaging/cropped_P1C02.png]

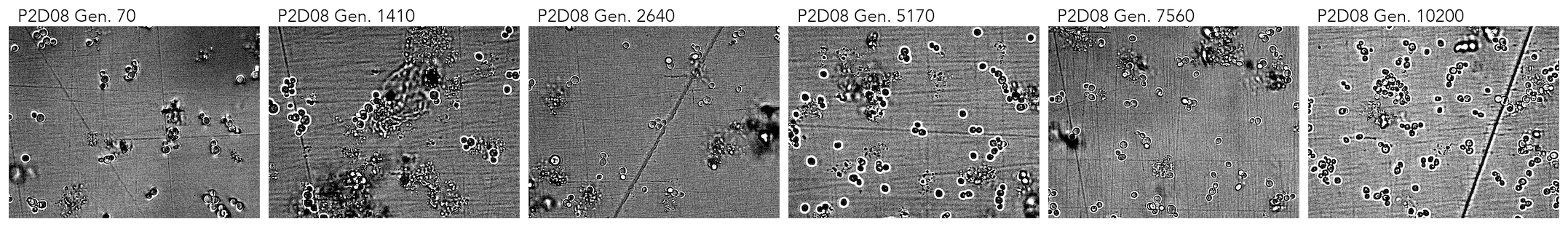

Supplement: Supplementary file 6. [file elife-63910-supp6.zip › imaging/cropped_P2D08.png]

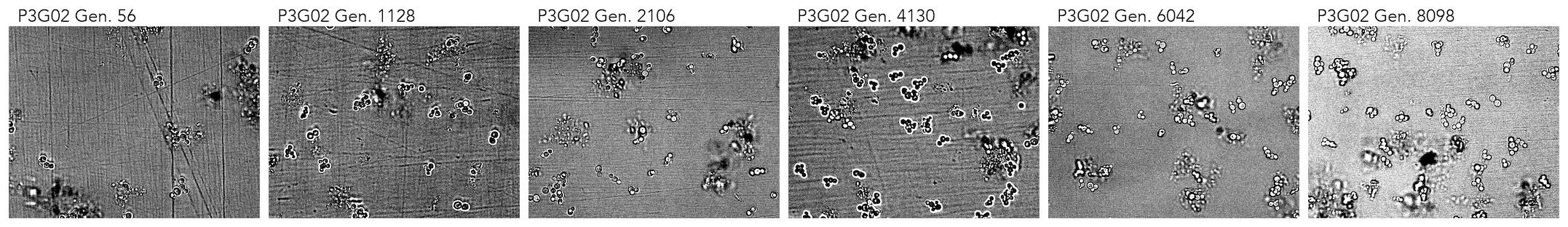

Supplement: Supplementary file 6. [file elife-63910-supp6.zip › imaging/cropped_P3G02.png]

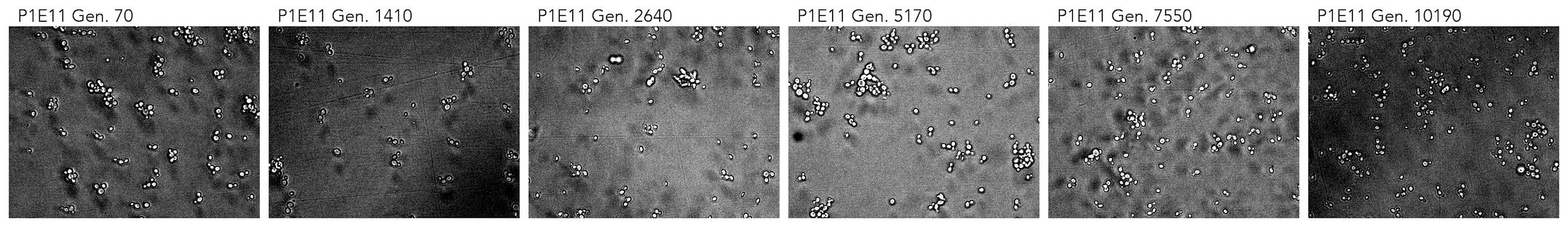

Supplement: Supplementary file 6. [file elife-63910-supp6.zip › imaging/cropped_P1E11.png]

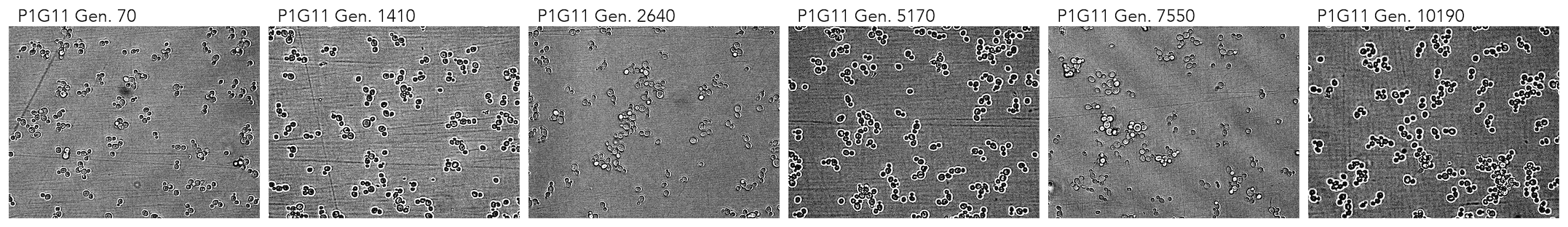

Supplement: Supplementary file 6. [file elife-63910-supp6.zip › imaging/cropped_P1G11.png]

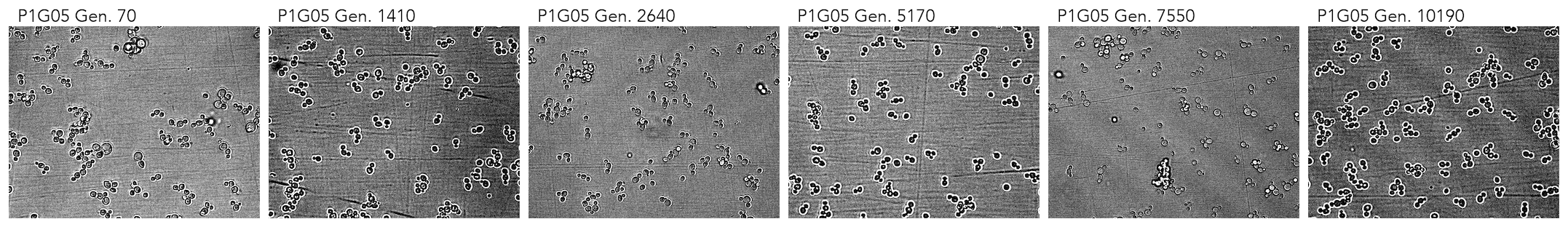

Supplement: Supplementary file 6. [file elife-63910-supp6.zip › imaging/cropped_P1G05.png]

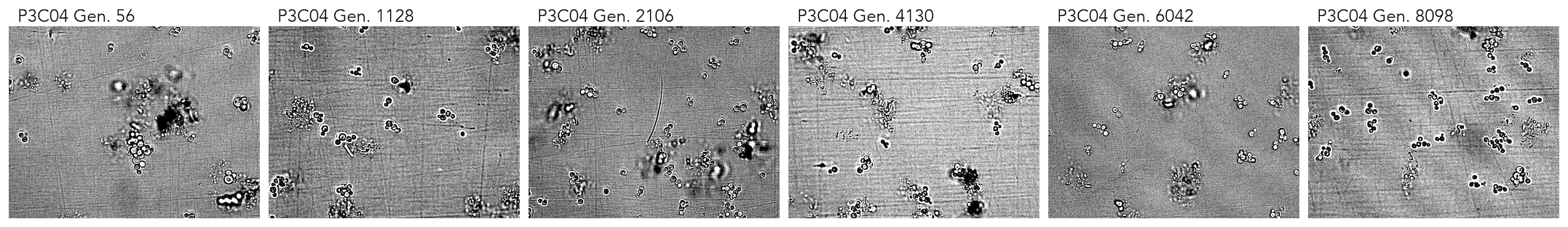

Supplement: Supplementary file 6. [file elife-63910-supp6.zip › imaging/cropped_P3C04.png]

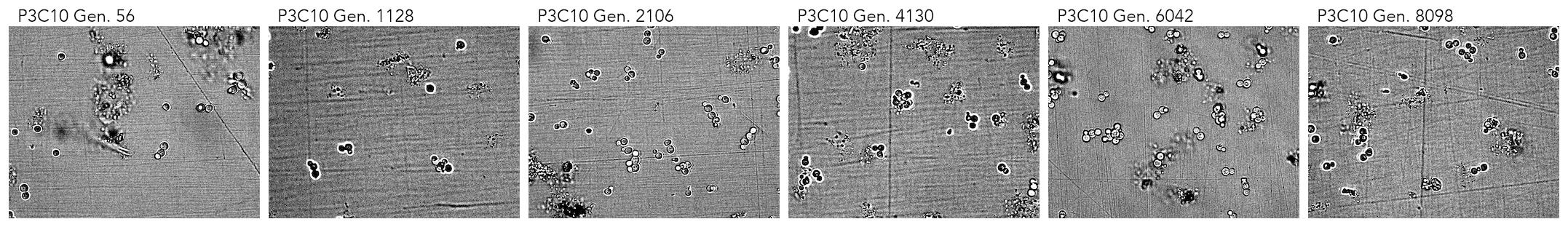

Supplement: Supplementary file 6. [file elife-63910-supp6.zip › imaging/cropped_P3C10.png]

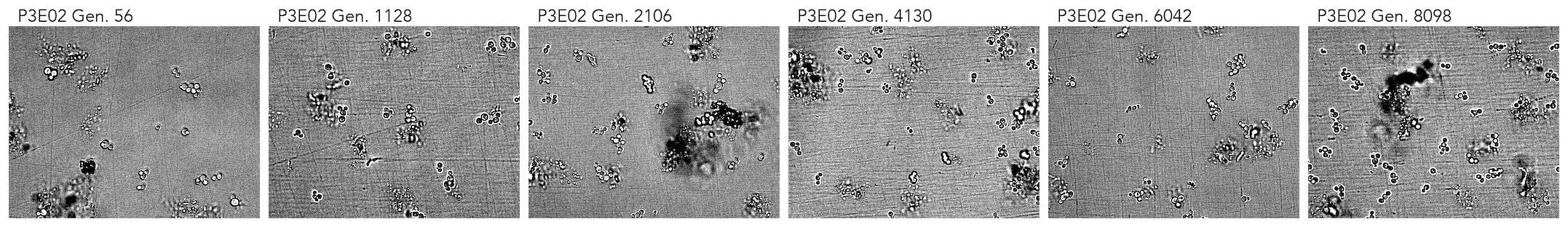

Supplement: Supplementary file 6. [file elife-63910-supp6.zip › imaging/cropped_P3E02.png]

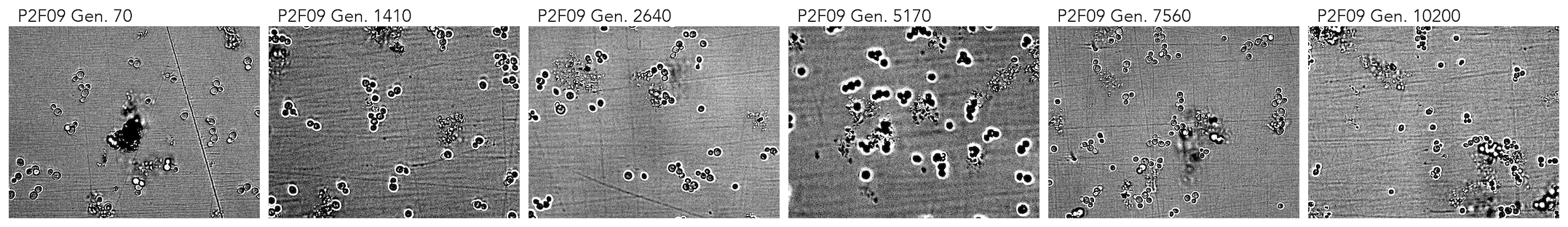

Supplement: Supplementary file 6. [file elife-63910-supp6.zip › imaging/cropped_P2F09.png]

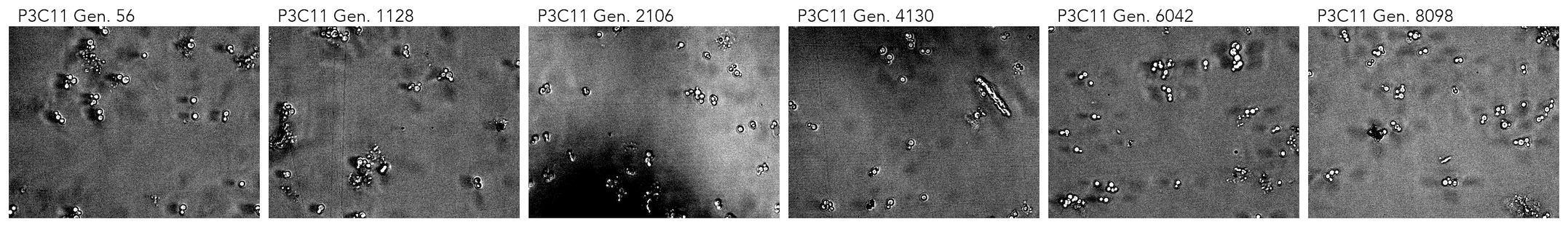

Supplement: Supplementary file 6. [file elife-63910-supp6.zip › imaging/cropped_P3C11.png]

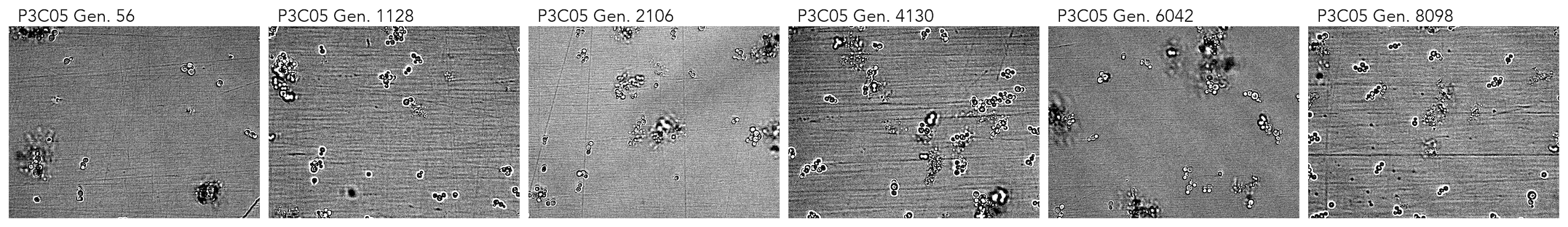

Supplement: Supplementary file 6. [file elife-63910-supp6.zip › imaging/cropped_P3C05.png]

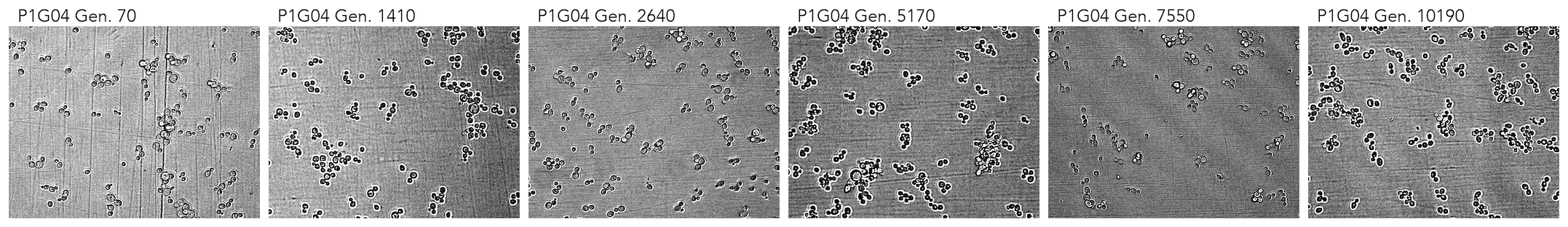

Supplement: Supplementary file 6. [file elife-63910-supp6.zip › imaging/cropped_P1G04.png]

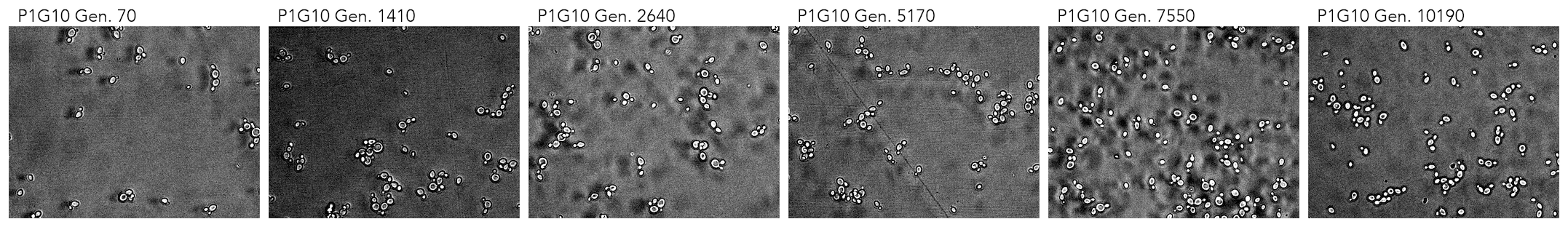

Supplement: Supplementary file 6. [file elife-63910-supp6.zip › imaging/cropped_P1G10.png]

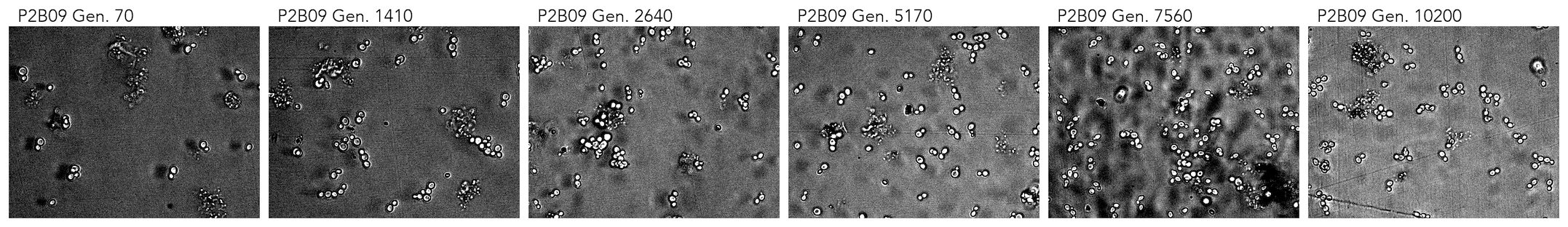

Supplement: Supplementary file 6. [file elife-63910-supp6.zip › imaging/cropped_P2B09.png]

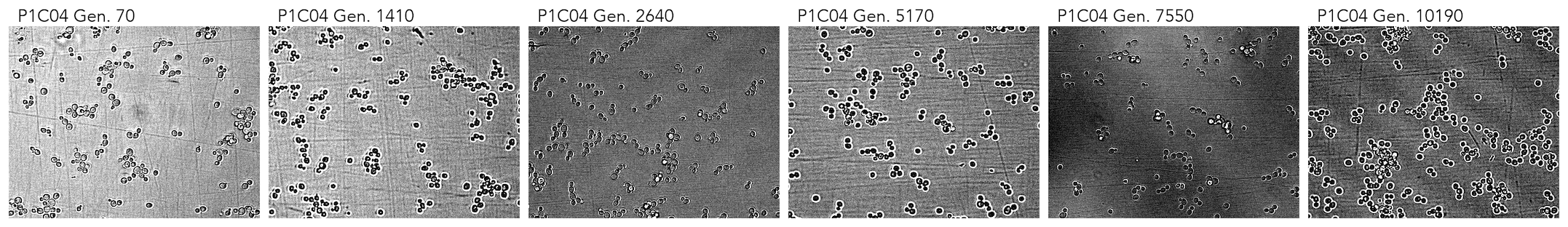

Supplement: Supplementary file 6. [file elife-63910-supp6.zip › imaging/cropped_P1C04.png]

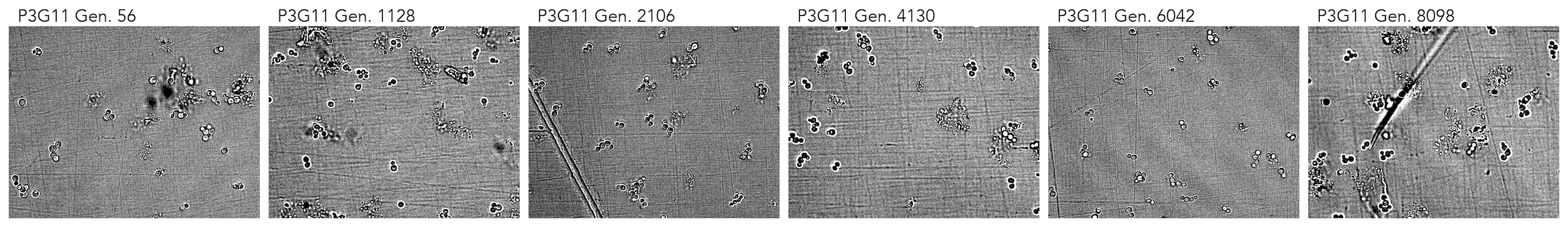

Supplement: Supplementary file 6. [file elife-63910-supp6.zip › imaging/cropped_P3G11.png]

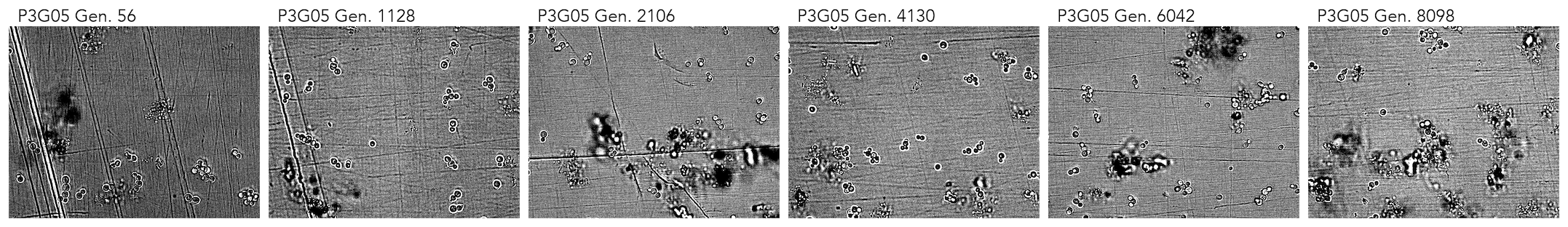

Supplement: Supplementary file 6. [file elife-63910-supp6.zip › imaging/cropped_P3G05.png]

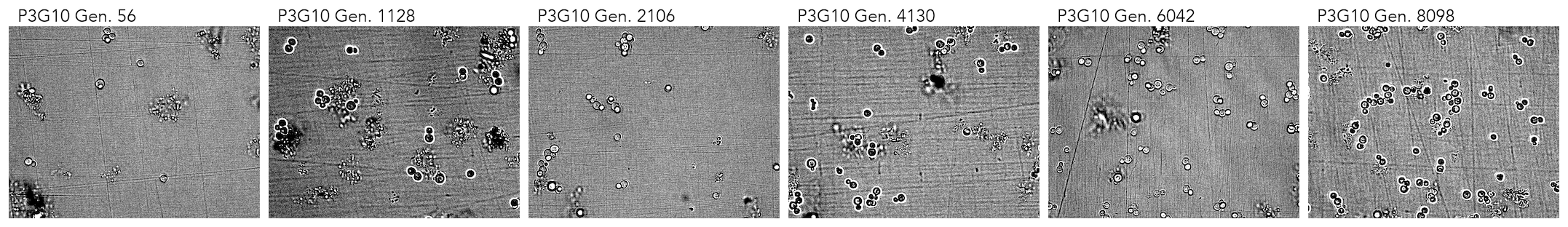

Supplement: Supplementary file 6. [file elife-63910-supp6.zip › imaging/cropped_P3G10.png]

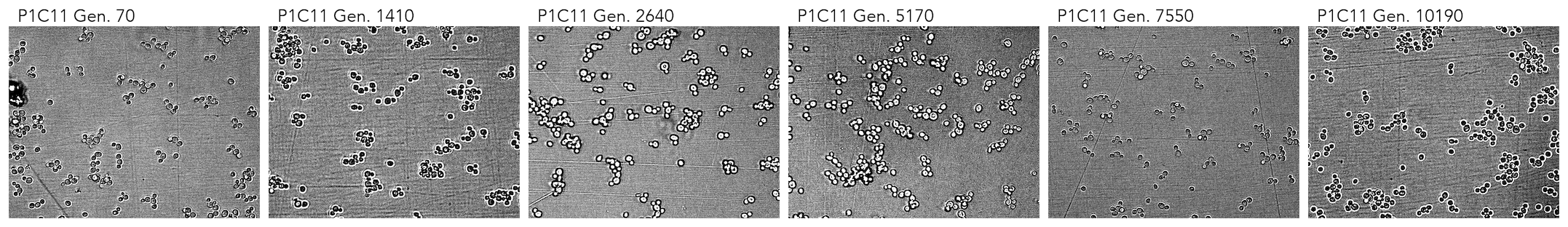

Supplement: Supplementary file 6. [file elife-63910-supp6.zip › imaging/cropped_P1C11.png]

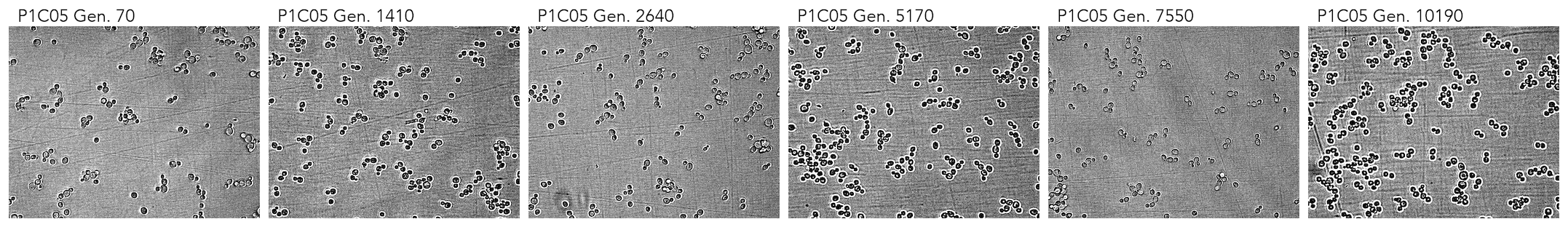

Supplement: Supplementary file 6. [file elife-63910-supp6.zip › imaging/cropped_P1C05.png]

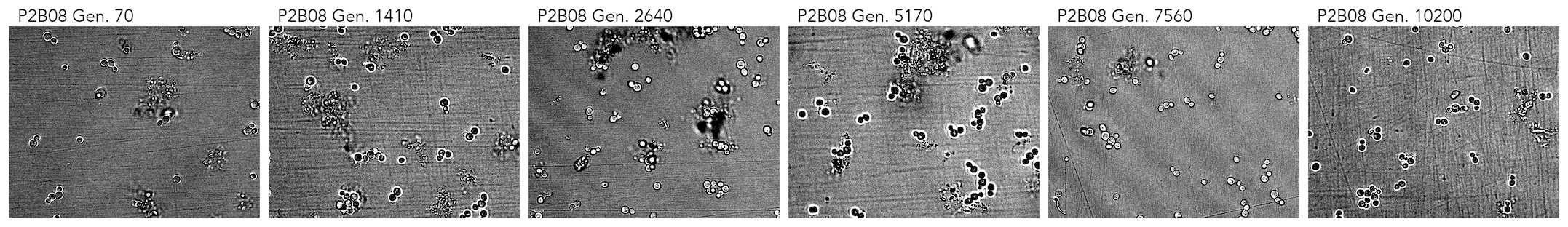

Supplement: Supplementary file 6. [file elife-63910-supp6.zip › imaging/cropped_P2B08.png]

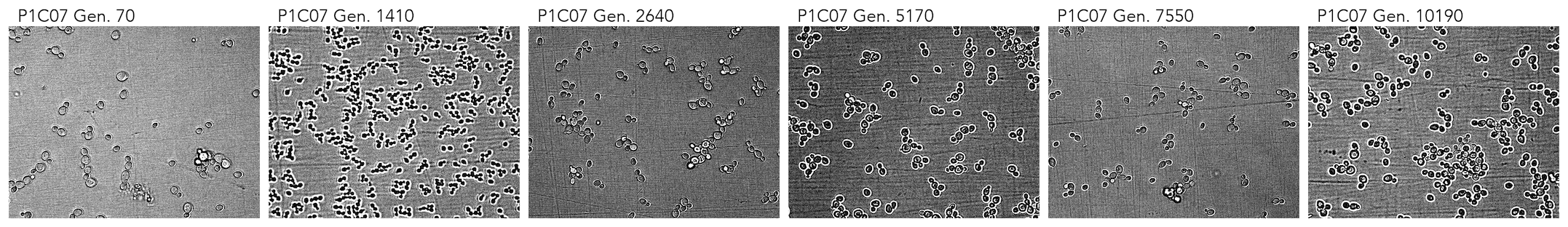

Supplement: Supplementary file 6. [file elife-63910-supp6.zip › imaging/cropped_P1C07.png]

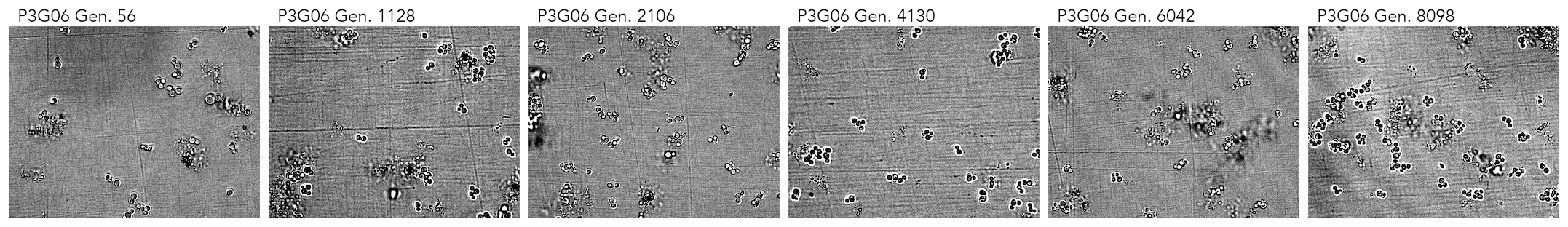

Supplement: Supplementary file 6. [file elife-63910-supp6.zip › imaging/cropped_P3G06.png]

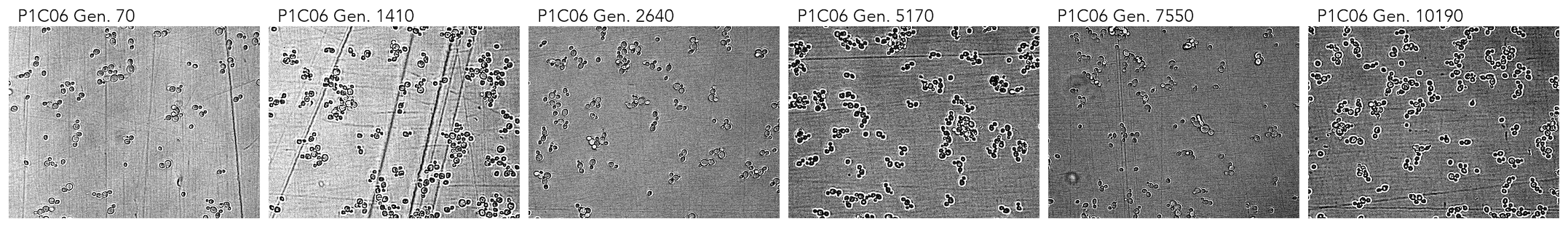

Supplement: Supplementary file 6. [file elife-63910-supp6.zip › imaging/cropped_P1C06.png]

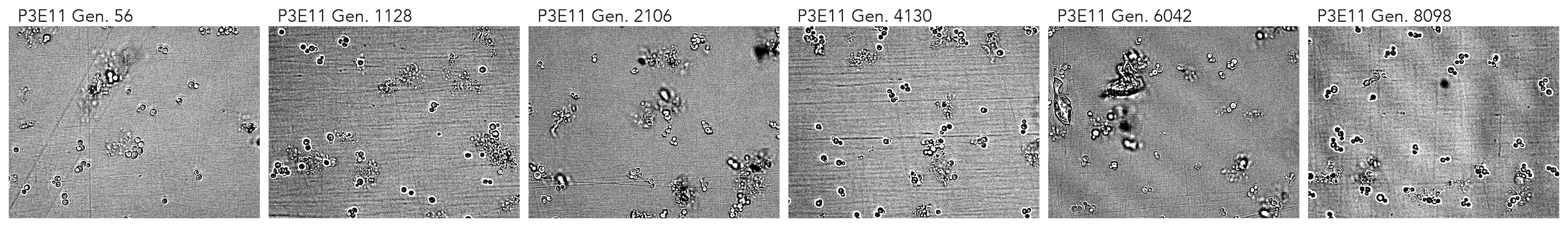

Supplement: Supplementary file 6. [file elife-63910-supp6.zip › imaging/cropped_P3E11.png]

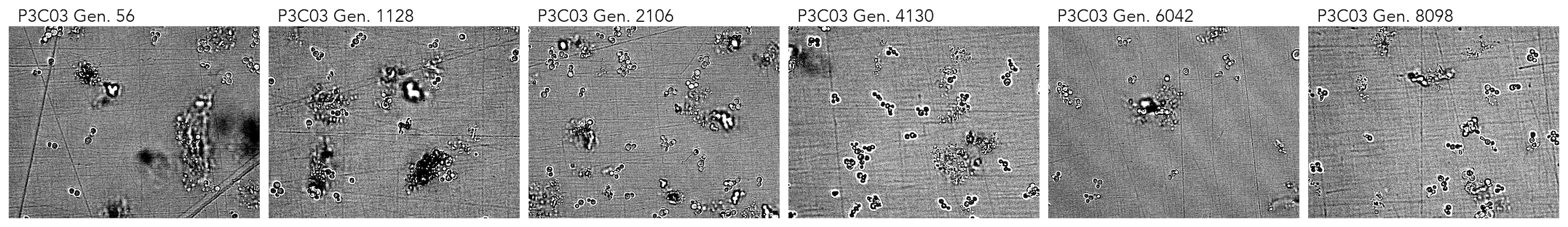

Supplement: Supplementary file 6. [file elife-63910-supp6.zip › imaging/cropped_P3C03.png]

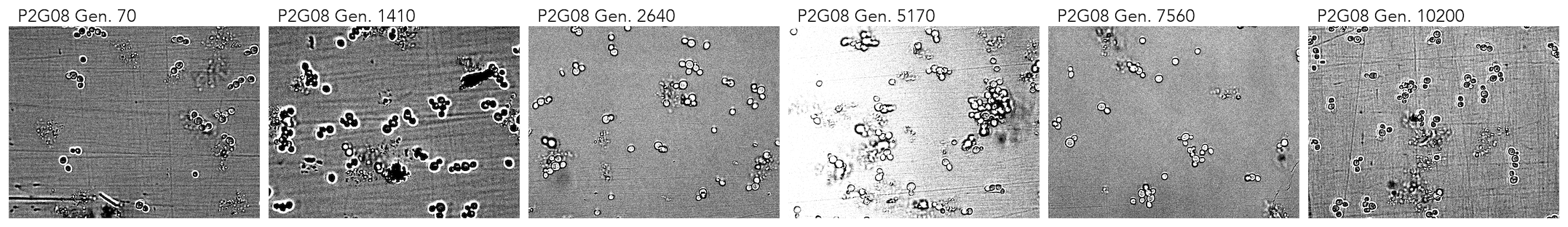

Supplement: Supplementary file 6. [file elife-63910-supp6.zip › imaging/cropped_P2G08.png]

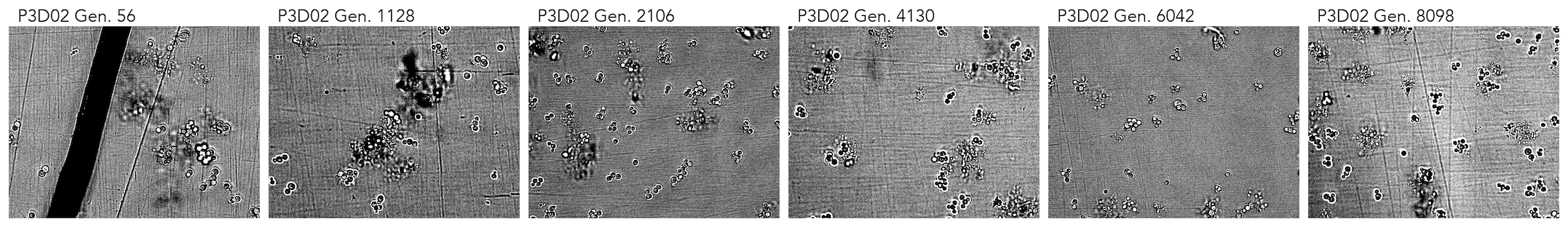

Supplement: Supplementary file 6. [file elife-63910-supp6.zip › imaging/cropped_P3D02.png]

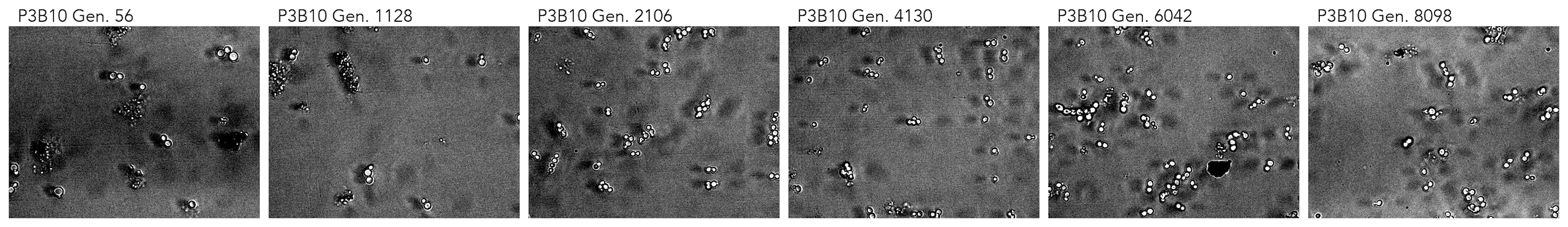

Supplement: Supplementary file 6. [file elife-63910-supp6.zip › imaging/cropped_P3B10.png]

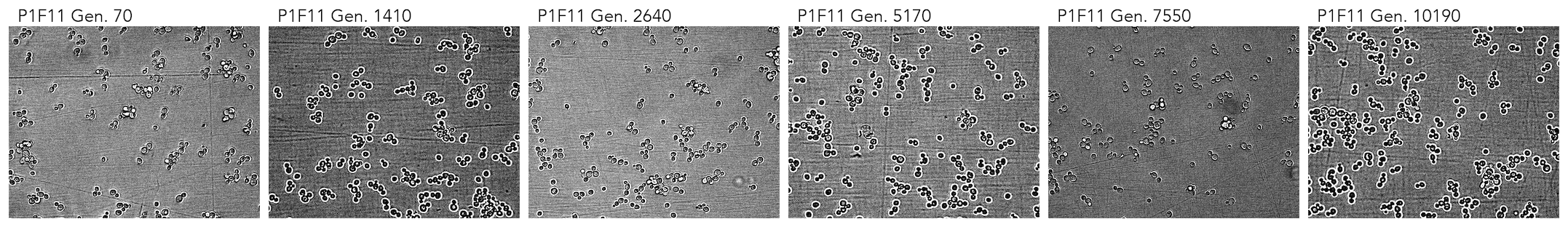

Supplement: Supplementary file 6. [file elife-63910-supp6.zip › imaging/cropped_P1F11.png]

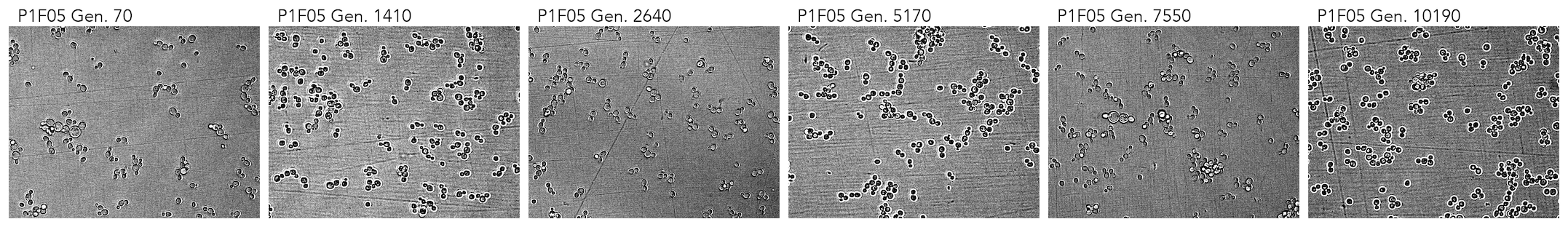

Supplement: Supplementary file 6. [file elife-63910-supp6.zip › imaging/cropped_P1F05.png]

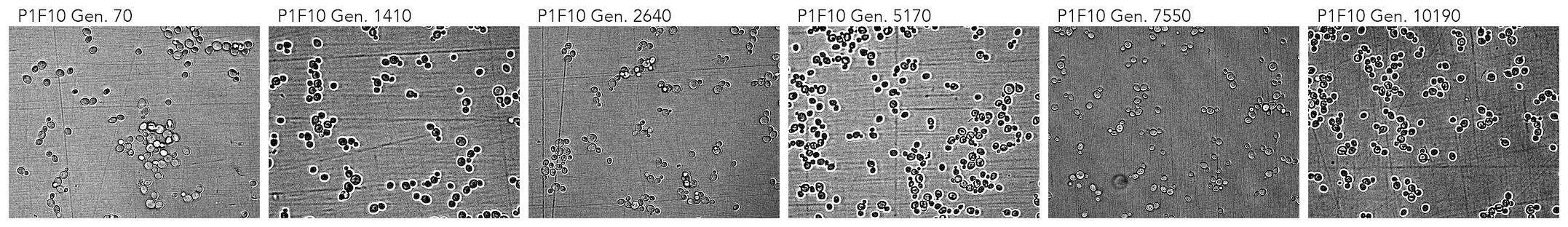

Supplement: Supplementary file 6. [file elife-63910-supp6.zip › imaging/cropped_P1F10.png]

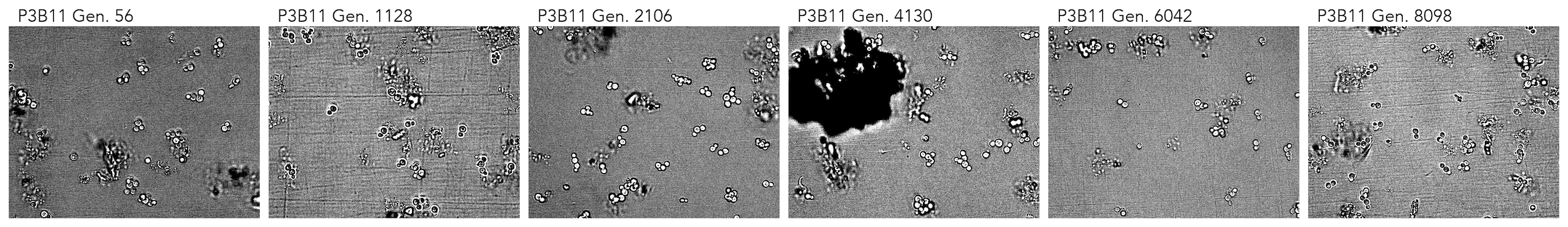

Supplement: Supplementary file 6. [file elife-63910-supp6.zip › imaging/cropped_P3B11.png]

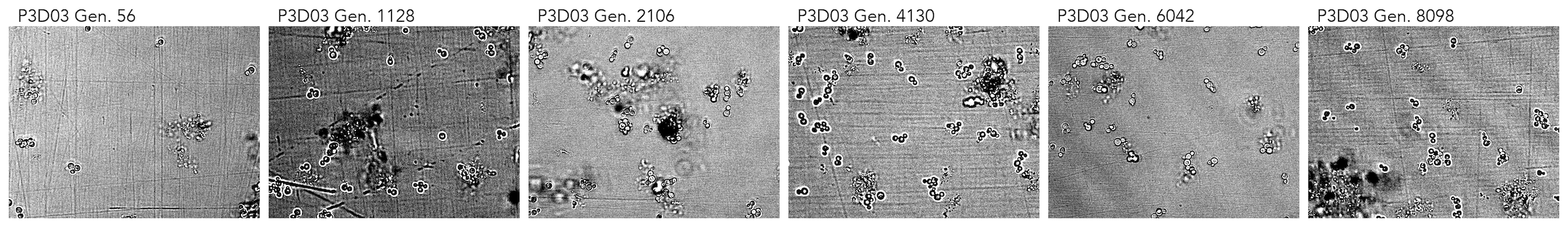

Supplement: Supplementary file 6. [file elife-63910-supp6.zip › imaging/cropped_P3D03.png]

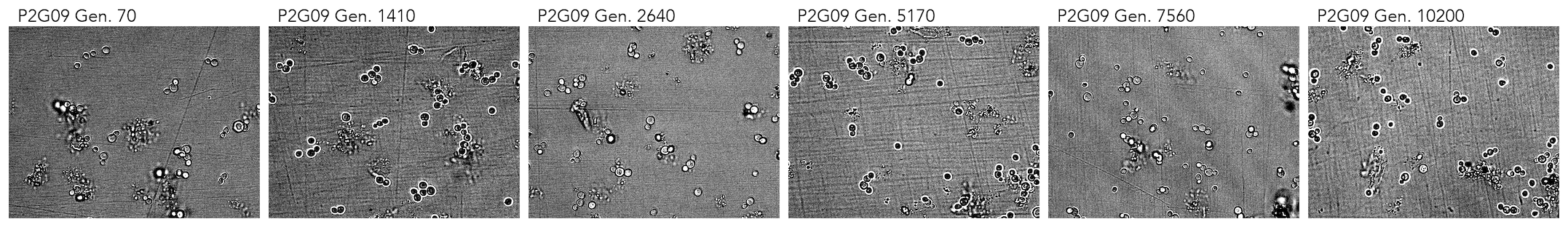

Supplement: Supplementary file 6. [file elife-63910-supp6.zip › imaging/cropped_P2G09.png]

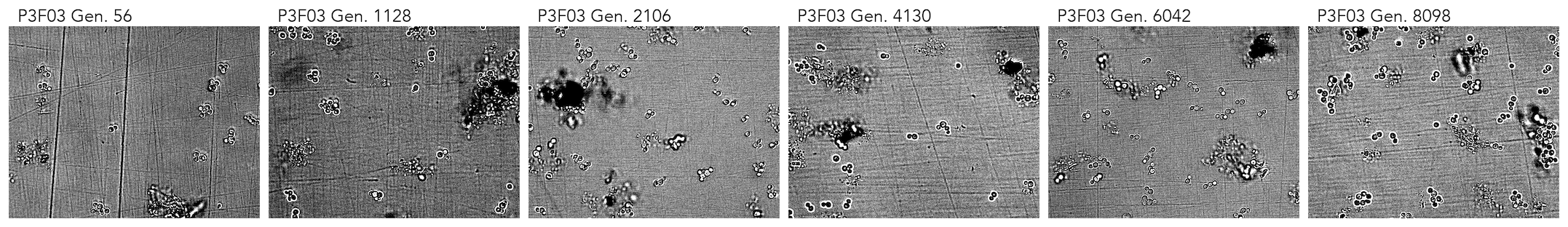

Supplement: Supplementary file 6. [file elife-63910-supp6.zip › imaging/cropped_P3F03.png]

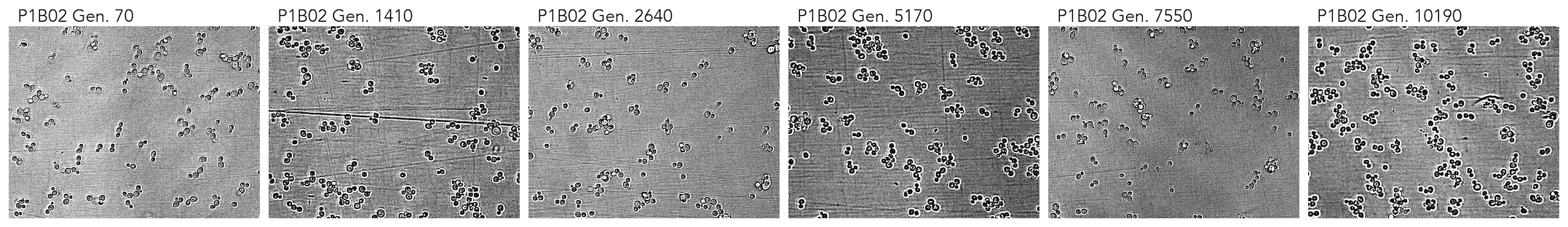

Supplement: Supplementary file 6. [file elife-63910-supp6.zip › imaging/cropped_P1B02.png]

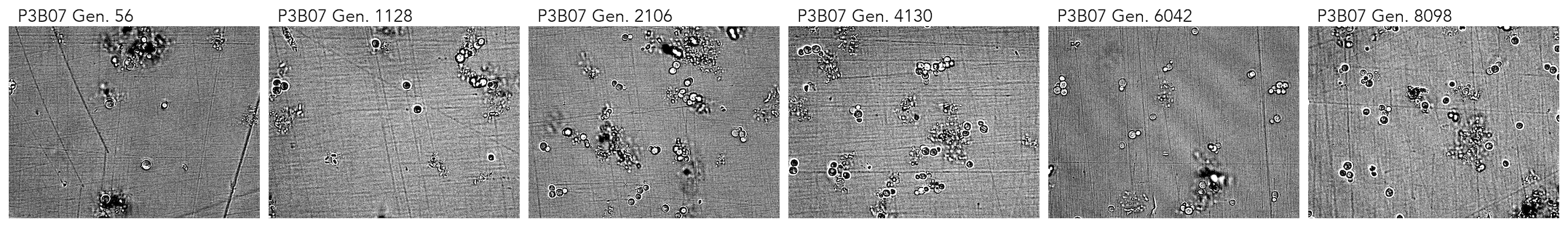

Supplement: Supplementary file 6. [file elife-63910-supp6.zip › imaging/cropped_P3B07.png]

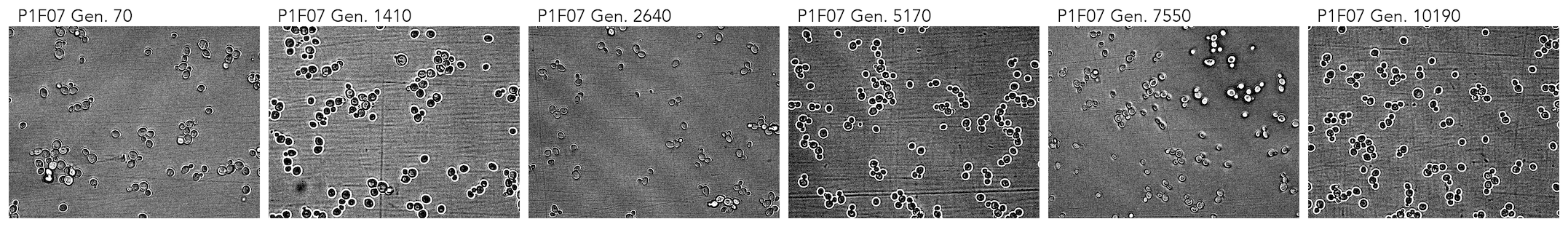

Supplement: Supplementary file 6. [file elife-63910-supp6.zip › imaging/cropped_P1F07.png]

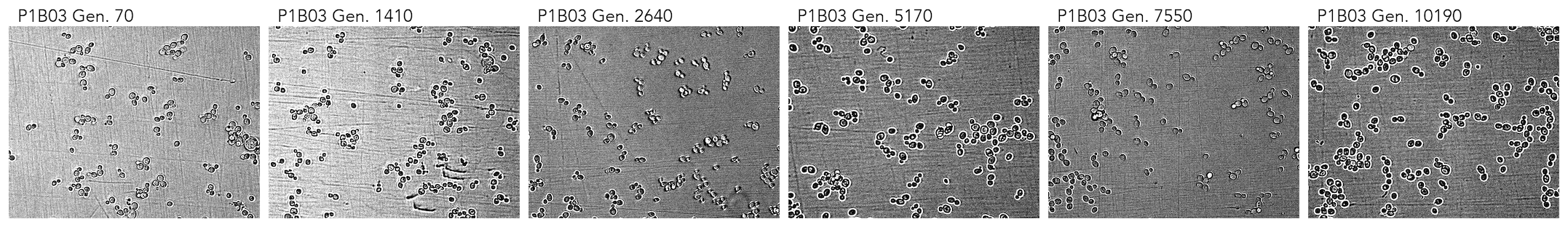

Supplement: Supplementary file 6. [file elife-63910-supp6.zip › imaging/cropped_P1B03.png]

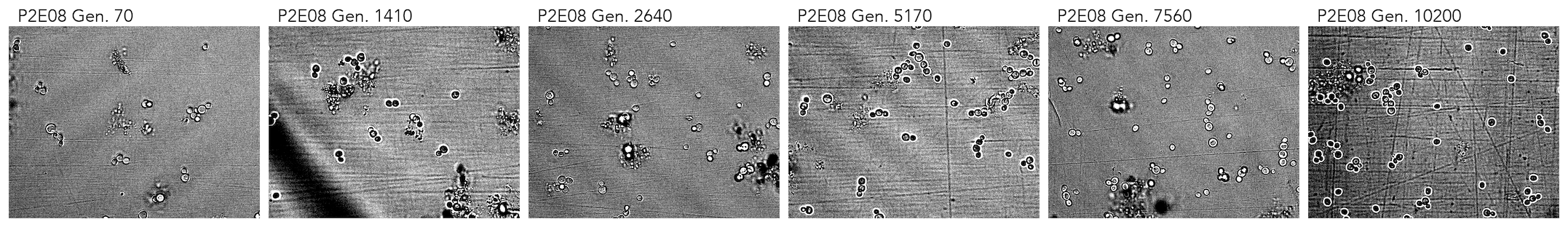

Supplement: Supplementary file 6. [file elife-63910-supp6.zip › imaging/cropped_P2E08.png]

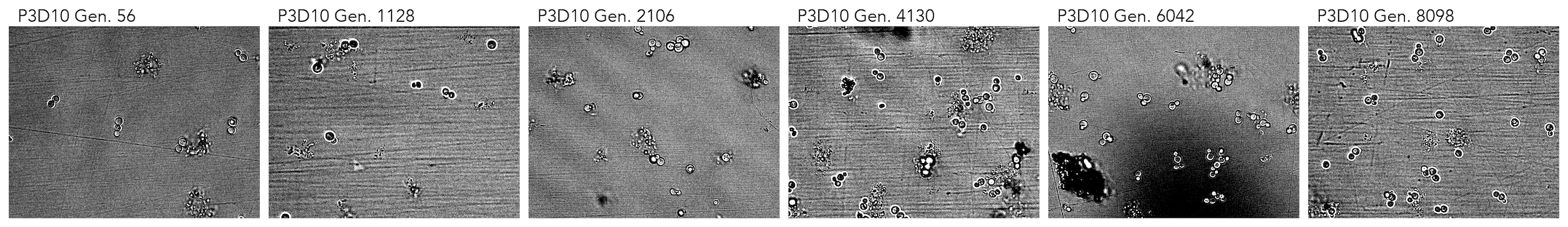

Supplement: Supplementary file 6. [file elife-63910-supp6.zip › imaging/cropped_P3D10.png]

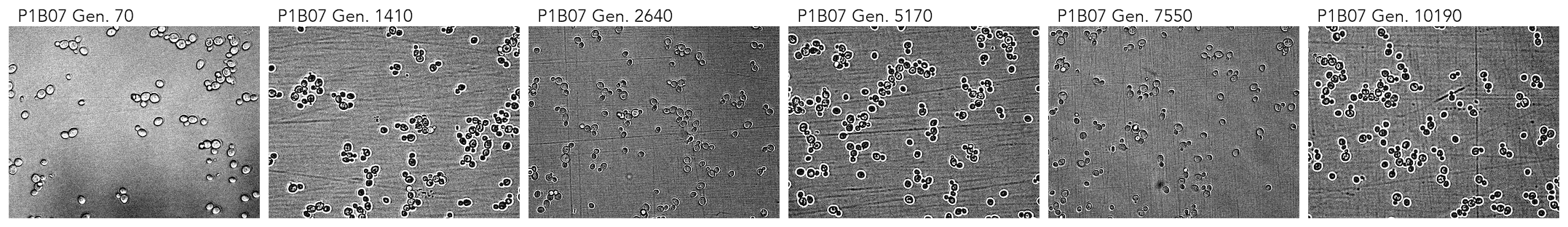

Supplement: Supplementary file 6. [file elife-63910-supp6.zip › imaging/cropped_P1B07.png]

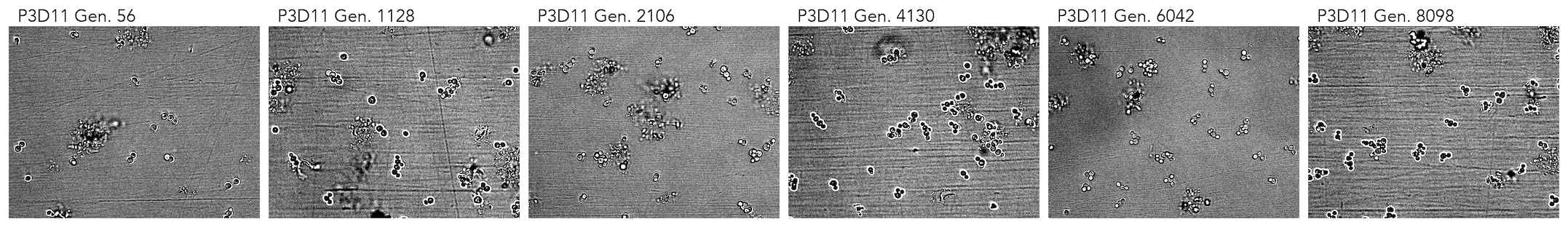

Supplement: Supplementary file 6. [file elife-63910-supp6.zip › imaging/cropped_P3D11.png]

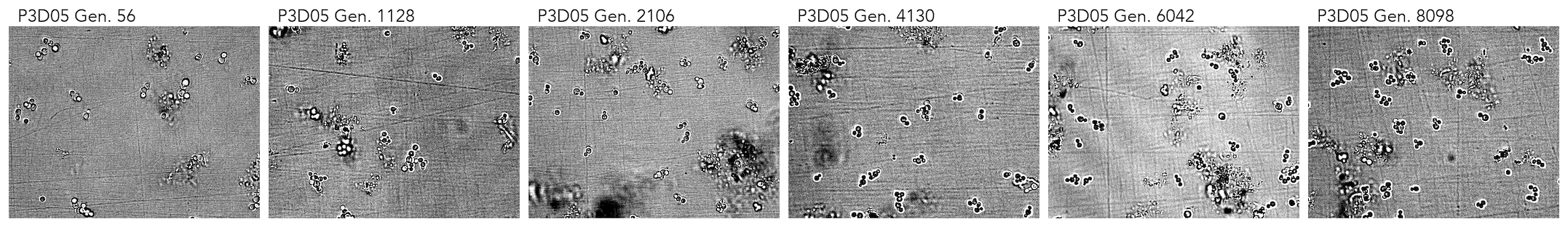

Supplement: Supplementary file 6. [file elife-63910-supp6.zip › imaging/cropped_P3D05.png]

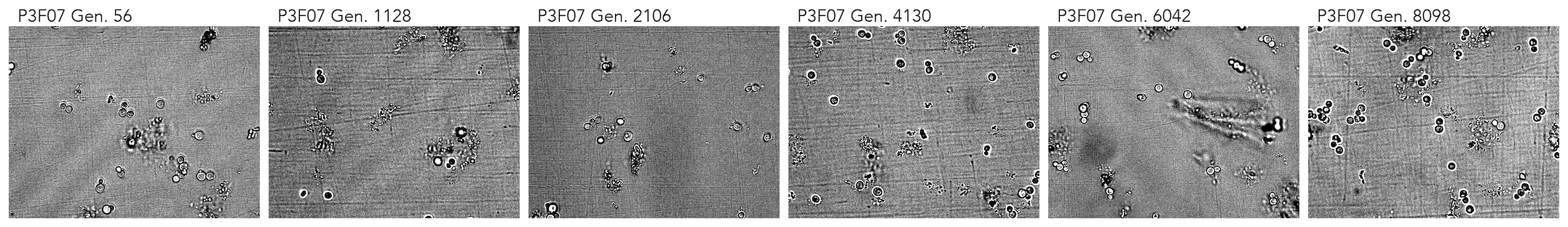

Supplement: Supplementary file 6. [file elife-63910-supp6.zip › imaging/cropped_P3F07.png]

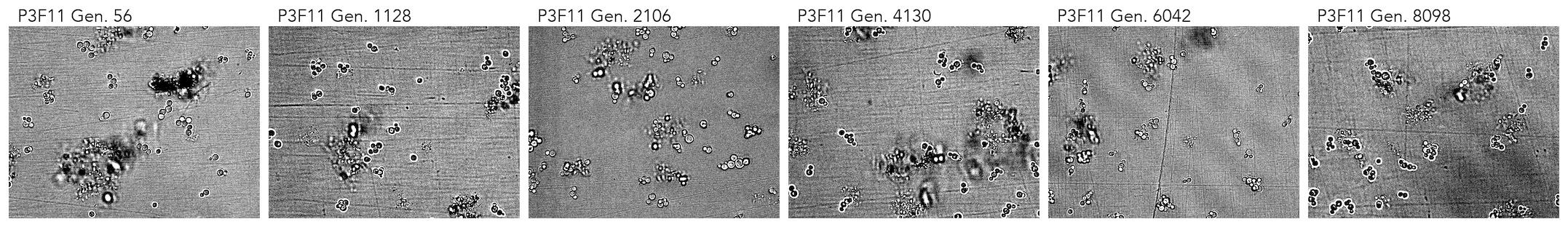

Supplement: Supplementary file 6. [file elife-63910-supp6.zip › imaging/cropped_P3F11.png]

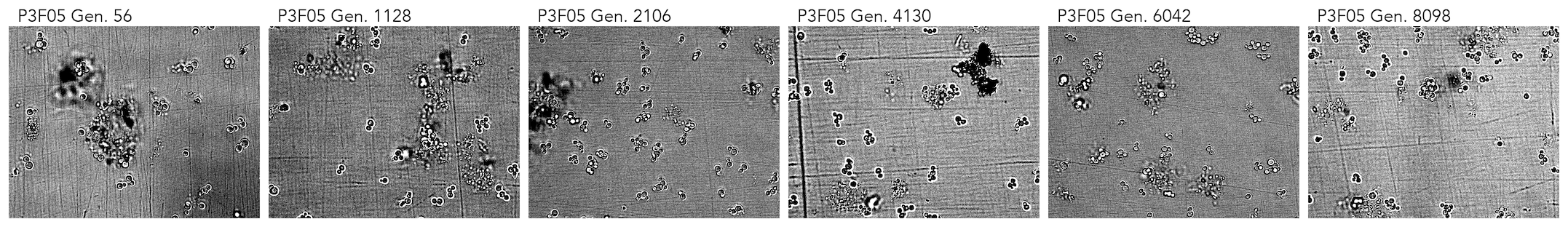

Supplement: Supplementary file 6. [file elife-63910-supp6.zip › imaging/cropped_P3F05.png]

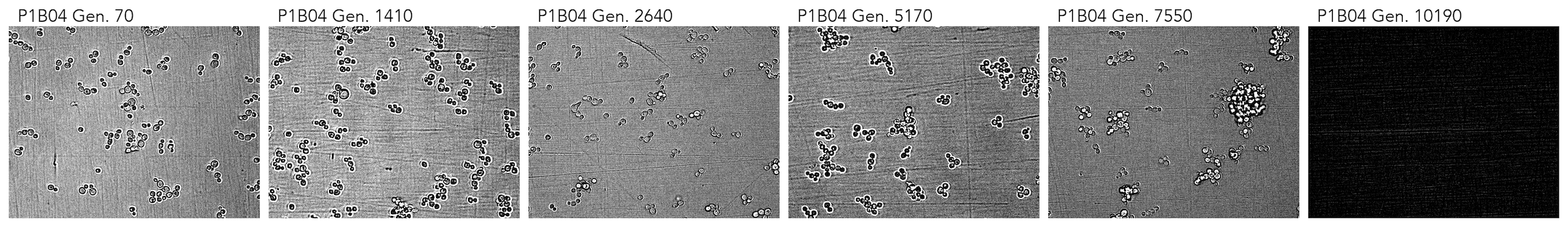

Supplement: Supplementary file 6. [file elife-63910-supp6.zip › imaging/cropped_P1B04.png]

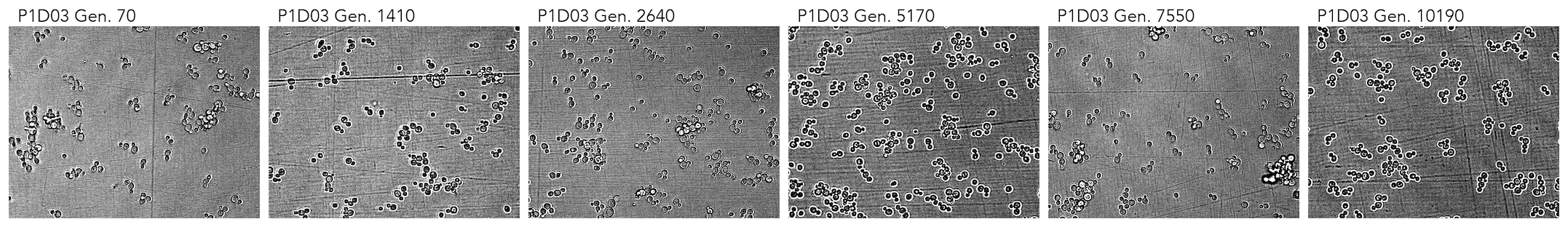

Supplement: Supplementary file 6. [file elife-63910-supp6.zip › imaging/cropped_P1D03.png]

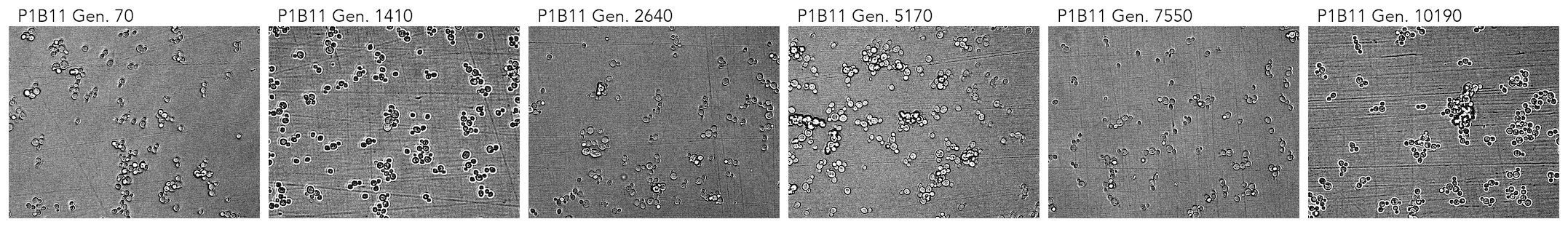

Supplement: Supplementary file 6. [file elife-63910-supp6.zip › imaging/cropped_P1B11.png]
