# Supplementary material for: Phenotypic and molecular evolution across 10,000 generations in laboratory budding yeast populations
Source: eLife. 2021 Jan 19;10:e63910. doi: 10.7554/eLife.63910 (PMC7815316; doi:10.7554/eLife.63910)
Supplement: Transparent reporting form [file elife-63910-transrepform.docx]

***eLife’s* transparent reporting form**

We encourage authors to provide detailed information *within their submission* to facilitate the interpretation and replication of experiments. Authors can upload supporting documentation to indicate the use of appropriate reporting guidelines for health-related research (see [EQUATOR Network](http://www.equator-network.org/%20)), life science research (see the [BioSharing Information Resource](https://biosharing.org/)), or the [ARRIVE guidelines](http://www.plosbiology.org/article/info:doi/10.1371/journal.pbio.1000412) for reporting work involving animal research. Where applicable, authors should refer to any relevant reporting standards documents in this form.

If you have any questions, please consult our Journal Policies and/or contact us: [editorial@elifesciences.org](mailto:editorial@elifesciences.org).

**Sample-size estimation**

- You should state whether an appropriate sample size was computed when the study was being designed
- You should state the statistical method of sample size computation and any required assumptions
- If no explicit power analysis was used, you should describe how you decided what sample (replicate) size (number) to use

Please outline where this information can be found within the submission (e.g., sections or figure legends), or explain why this information doesn’t apply to your submission:

This is not applicable to our submission (this is an experimental evolution study, no power analysis or sample size estimation would be possible or appropriate).

**Replicates**

- You should report how often each experiment was performed
- You should include a definition of biological versus technical replication
- The data obtained should be provided and sufficient information should be provided to indicate the number of independent biological and/or technical replicates
- If you encountered any outliers, you should describe how these were handled
- Criteria for exclusion/inclusion of data should be clearly stated
- High-throughput sequence data should be uploaded before submission, with a private link for reviewers provided (these are available from both GEO and ArrayExpress)

Please outline where this information can be found within the submission (e.g., sections or figure legends), or explain why this information doesn’t apply to your submission:

We describe the number of evolution populations in each environment-strain combination in the first paragraph of the results, and list those that were lost or excluded in Figure 1 – figure supplement 1. We note the number of fitness assay replicates in the corresponding methods section. The only other population excluded from analysis is P1B03, a haploid population that becomes diploid, and we discuss this in the main text (“Patterns of molecular evolution specific to diploids” paragraph 1) and in Figure 8 – figure supplement 1. We describe the number of populations sequenced in “Molecular evolution”, paragraph 1, and describe how mutations were filtered in the final variant calls in the “Sequencing analysis” section of the Methods. We uploaded Fastq files from this project to the GenBank SRA (accession: SRP286889).

**Statistical reporting**

- Statistical analysis methods should be described and justified
- Raw data should be presented in figures whenever informative to do so (typically when N per group is less than 10)
- For each experiment, you should identify the statistical tests used, exact values of N, definitions of center, methods of multiple test correction, and dispersion and precision measures (e.g., mean, median, SD, SEM, confidence intervals; and, for the major substantive results, a measure of effect size (e.g., Pearson's r, Cohen's d)
- Report exact p-values wherever possible alongside the summary statistics and 95% confidence intervals. These should be reported for all key questions and not only when the p-value is less than 0.05.

Please outline where this information can be found within the submission (e.g., sections or figure legends), or explain why this information doesn’t apply to your submission:

Statistical methods are described in all subsections of the Methods text, and all analysis code is available at <https://github.com/mjohnson11/VLTE_PIPELINES> . We annotated all comparisons between rates of fitness gain and dN/dS ratios of different strains with the test used (Mann-Whitney U test), and the P-value and N per group for all of these tests is recorded in Supplementary file 1. The statistical tests used to examine multi-hit gene enrichment by strain and/or environment, mutual information between genes, and mutation overdispersion are described in the corresponding three sections of the methods text. All three of these methods utilize simulated null datasets, which are also described in these sections. The results and p-values for these tests are available in Supplementary file 4, Figure 7 – figure supplement 1, and Figure 7 – figure supplement 2, respectively.

(For large datasets, or papers with a very large number of statistical tests, you may upload a single table file with tests, Ns, etc., with reference to sections in the manuscript.)

**Group allocation**

- Indicate how samples were allocated into experimental groups (in the case of clinical studies, please specify allocation to treatment method); if randomization was used, please also state if restricted randomization was applied
- Indicate if masking was used during group allocation, data collection and/or data analysis

Please outline where this information can be found within the submission (e.g., sections or figure legends), or explain why this information doesn’t apply to your submission:

This is an experimental evolution project, so we did not have experimental groups or use masking. We started replicate evolution populations from randomly chosen clones and chose focal populations randomly, as described in the Methods.

**Additional data files (“source data”)**

- We encourage you to upload relevant additional data files, such as numerical data that are represented as a graph in a figure, or as a summary table
- Where provided, these should be in the most useful format, and they can be uploaded as “Source data” files linked to a main figure or table
- Include model definition files including the full list of parameters used
- Include code used for data analysis (e.g., R, MatLab)
- Avoid stating that data files are “available upon request”

Please indicate the figures or tables for which source data files have been provided:

All analysis code is available at <https://github.com/mjohnson11/VLTE_PIPELINES> .

The data used to produce Figure 2 and 9A are available in Supplementary file 1. The data used to produce Figure 3, 4, 5, 6, 8, and 9B are available in Supplementary file 2 and Supplementary file 4. The data used to produce the Figure supplements are the same as that used to produce the corresponding main figure, except in these cases: Figure 3 – figure supplements 11-12 correspond to Supplementary file 3 (and the sequencing data posted on SRA), the data for Figure 4 – figure supplement 1 is in Supplementary file 5, and the data from Figure 8 – figure supplement 2 is from Supplementary file 6.
